# Experimental and evolutionary evidence for horizontal transfer of an envelope fusion protein gene between thogotoviruses and baculoviruses

**DOI:** 10.1128/jvi.02148-24

**Published:** 2025-06-25

**Authors:** Bruno Milhomem Pilati Rodrigues, Luis Janssen, Leonardo Assis da Silva, Suzane Suliane Vitorino Gomes Acacio, Mariana Tigano Magalhães, Bergmann Morais Ribeiro

**Affiliations:** 1 Cell Biology Department, Universidade de Brasília 28127, Brasília, Brazil; Wageningen University & Research, Wageningen, Netherlands

**Keywords:** horizontal gene transfer, baculovirus GP64 protein, thogotovirus fusion proteins, viral evolution and phylogenetics, biotechnological applications in gene delivery

## Abstract

Baculoviruses are insect-specific viruses with large, double-stranded DNA genomes classified into four genera. Alphabaculoviruses, which infect lepidoptera, are further divided into group I (G1-α) and group II (G2-α). The GP64 protein, essential for cell attachment and viral entry in G1-α baculoviruses, is thought to have originated through horizontal gene transfer (HGT) from thogotoviruses (family *Orthomyxoviridae*). This study investigates the functional substitution of GP64 by thogotovirus fusion proteins. Through RNA-seq data mining, we identified a novel thogotovirus, Melitaea didyma thogotovirus 1 (MediTHOV-1), in lepidopteran hosts. Phylodynamic analysis of G1-α baculovirus and thogotovirus glycoproteins suggests that the HGT event occurred during the Mesozoic era. To test functional substitution, we constructed recombinant Autographa californica multiple nucleopolyhedrovirus (AcMNPV) carrying either the envelope fusion protein (EFP) genes from MediTHOV-1 or Apis thogotovirus 1 (ATHOV-1), while deleted for its native *gp64* gene. Our results show that, while the MediTHOV-1 glycoprotein failed to rescue AcMNPV infectivity, the ATHOV-1 fusion protein (EFP) partially restored infectivity, albeit with reduced efficiency. Cryo-electron microscopy revealed lower incorporation of ATHOV-1 EFP into viral envelopes compared to GP64. The recombinant AcMNPV carrying ATHOV-1 EFP (Ac-ATHOVGPgp64Δ) displayed delayed replication kinetics and lower viral titers. Interestingly, ATHOV-1 EFP significantly enhanced baculovirus entry and gene transduction in mosquito cells. These findings provide experimental support for the HGT hypothesis, demonstrating the functional incorporation of a thogotovirus glycoprotein into a baculovirus. This study sheds light on the evolutionary relationship between baculovirus GP64 and glycoproteins, offering insights into viral evolution and potential biotechnological applications in gene delivery and protein expression.

**IMPORTANCE:**

Baculoviruses are widely utilized for the biological control of insect pests and as versatile biotechnological tools, with their effectiveness largely dependent on the activity of their envelope fusion proteins (EFPs). Thogotoviruses, in contrast, are emerging vector-borne pathogens of significant concern. In this study, we present the first successful functional substitution of the baculovirus GP64 protein with a thogotovirus EFP, alongside the identification of what appears to be a lepidopteran-associated thogotovirus, Melitaea didyma thogothovirus 1. Our work provides functional and phylogenetic insights into the evolutionary relationship between these distantly related viral groups, particularly the hypothesized horizontal gene transfer event that gave rise to baculoviral *gp64* gene. These findings offer a deeper understanding of the determinants underlying the adaptation of baculoviral glycoproteins to novel hosts. Furthermore, the discovery of novel viral genes highlights promising opportunities for biotechnological advancements, including the development of enhanced baculovirus-based gene delivery systems and tools for protein expression.

## INTRODUCTION

Baculoviruses are insect-specific viruses with large double-stranded DNA genomes that contain numerous genes derived from exogenous sources, including their hosts and of other co-infecting viruses (1, 2). These genes have come to perform a variety of fundamental functions such as control of host physiology (3, 4) and modulation of apoptosis (5, 6), contributing to a complex and rich evolutionary history. The *Baculoviridae* family is divided into four genera, whose members infect insects in different orders: members of the *Alphabaculovirus* and *Betabaculovirus* genera infect lepidopterans, *Gammabaculovirus* members infect hymenoptera, while *Deltabaculovirus* members infect dipterans. Alphabaculoviruses can be phylogenetically further divided into group I (G1-α) and group II (G2-α) (7). During the replication of alphabaculoviruses and betabaculoviruses in susceptible insects, two viral phenotypes are produced: the budded virus (BV), which is responsible for cell-to-cell spread of infection within insect larvae, and the occlusion-derived virus (ODV), which is responsible for the infection from insect to insect (8). The major envelope fusion protein of G1-α baculoviruses is GP64 (herein referred to simply as GP64), present on the surface of the BV phenotype, while the F (fusion) protein is the major EFP of G2-α and betabaculoviruses (9). These proteins are crucial for cell attachment, viral entry, and efficient budding from infected insect cells (8, 10, 11).

The *Thogotovirus* genus belongs to the *Orthomyxoviridae* family, a group of segmented negative single-stranded RNA viruses, the latter being widely known for including the influenza viruses (12). Most thogotovirus members have been found in ticks, while a minority of thogotoviruses have also been isolated from mosquitos (13–16). Albeit this host representation may be biased toward vectors of viral diseases (17), some representatives of the genus, such as *Thogotovirus thogoto* (THOV) and viruses from the Dhori group, have been associated with zoonotic diseases, experimental infections in mice and naturally infected humans, including fatal human cases (16, 18–20), raising concerns about zoonotic outbreaks of thogotovirus infection. Their genomes are usually composed of six segments: three coding for subunits of an RNA-dependent RNA polymerase complex (PB2, PB1, and PA) and three coding for structural components (nucleocapsid protein NP, matrix protein M, and glycoprotein GP).

GP64 is structurally related to the class III viral fusion proteins, such as the G proteins of rhabdoviruses, the gB proteins of herpesviruses, and the glycoproteins (GPs) of thogotoviruses (21–23). Previous studies suggest that the GP64 of baculoviruses may have originated from a horizontal gene transfer (HGT) event from a thogotovirus (24, 25). This hypothesis is supported by the sequence homology and structural similarity between the envelope fusion proteins of these groups (Fig. S1) (24, 25). Additionally, GP-based phylogenies of thogotoviruses consistently group these viruses with G1-α baculoviruses, more closely than with other members of the *Orthomyxoviridae* family, with Sinu virus clustering with baculoviral sequences (25). Therefore, if this horizontal gene transfer event occurred, one could hypothesize that extant thogotovirus fusion proteins could remain functional within the context of a baculovirus infection, potentially substituting GP64 in extant baculoviruses for entry into susceptible insect cells.

To test this possibility, previous research has shown that the fusion proteins of Thogoto virus (26, 27) and Dhori virus (27), when incorporated into the genome of a baculovirus, could not rescue the infectivity of the baculovirus Autographa californica multiple nucleopolyhedrovirus (AcMNPV) when its native *gp64* gene was deleted.

We hypothesize that if an HGT event occurred, it likely took place during co-infection of a shared host between alphabaculoviruses and orthomyxoviruses. A recently identified thogotovirus, tentatively named Apis thogotovirus 1 (ATHOV-1) or Varroa orthomyxovirus-1 (VOV-1), has been detected in both *Apis mellifera* and the ectoparasitic mite *Varroa destructor* (28–30). Importantly, ATHOV-1 sequences were found in metagenome data sets from *A. mellifera* samples that lacked any reads corresponding to *V. destructor* or other common ectoparasites (28), indicating that bees themselves are likely the primary hosts of this virus. These findings support the hypothesis that ATHOV-1 is a bee-associated virus capable of infecting other invertebrates. These ecological overlaps with lepidopteran hosts may have facilitated the proposed gene transfer event. Furthermore, we utilized data mining in previously published RNA-seq data of the lepidopteran *Melitaea didyma* to describe the genome of a novel thogotovirus. Then, we performed phylodynamic analysis to estimate when this HGT event likely occurred.

To test the hypothesis that an insect thogotovirus fusion protein could functionally substitute GP64, we constructed recombinant AcMNPV lacking GP64 and containing the envelope glycoprotein gene of either Melitaea didyma thogotovirus 1 or Apis thogotovirus 1 (ATHOV-1) (28) and analyzed their infectivity in insect cells. We also evaluated the incorporation of EFPs into the viral envelope by cryo-electron microscopy (cryo-EM) and its effects in AcMNPV transduction efficiency *in vitro*.

## RESULTS

### Melitaea didyma thogotovirus 1 (MediTHOV-1), a putative novel lepidopteran infecting thogotovirus

We first searched for thogotoviruses in lepidoptera-derived sequencing reads, by searching in the Serratus web platform (31) for RNA-dependent RNA polymerase motifs closely related to that of PB1 of Sinu virus. In doing so, we identified a near-complete and novel genome with six segments of a lepidopteran orthomyxovirus evolutionarily close to thogotovirus. This virus was named Melitaea didyma thogotovirus 1 (MediTHOV-1). All segments showed an average read coverage greater than twenty. The genome comprises a total of 10,634 nucleotides, with the smallest segment containing 810 nucleotides (segment 6) and the largest containing 2,310 nucleotides (segment 1) (Table 1). The segments displayed conserved terminal sequences, specifically 3′AAAAA[T/C]CTCTTTGTTACTACTCCCG and 5′AGTAGTAACAAGAG[G/A]TTA.

**TABLE 1:** General characteristics of the genome of Melitaea didyma thogotovirus 1 (MediTHOV-1)

Genome segment, minimum coverage	Length (nts)	Length of 5′ UTR	ORF (nts)	ORF (AAs)	Length 3′ UTR	Thogothovirus homologous protein
S1, 22×	2,378	36	2,310 (37–2346)	769	32	PB2
S2, 46×	2,235	56	2,136 (57–2192)	711	43	PB1
S3, 59×	1,995	80	1,902 (81–1982)	633	13	PA
S4, 55×	1,419	29	1,380 (30–1409)	459	10	GP
S5**,** 26×	1,556	50	1,416 (51–1466)	471	90	NP
S6, 53×	1,051	79	810 (80–889)	269	162	M-like

**Fig 1:**
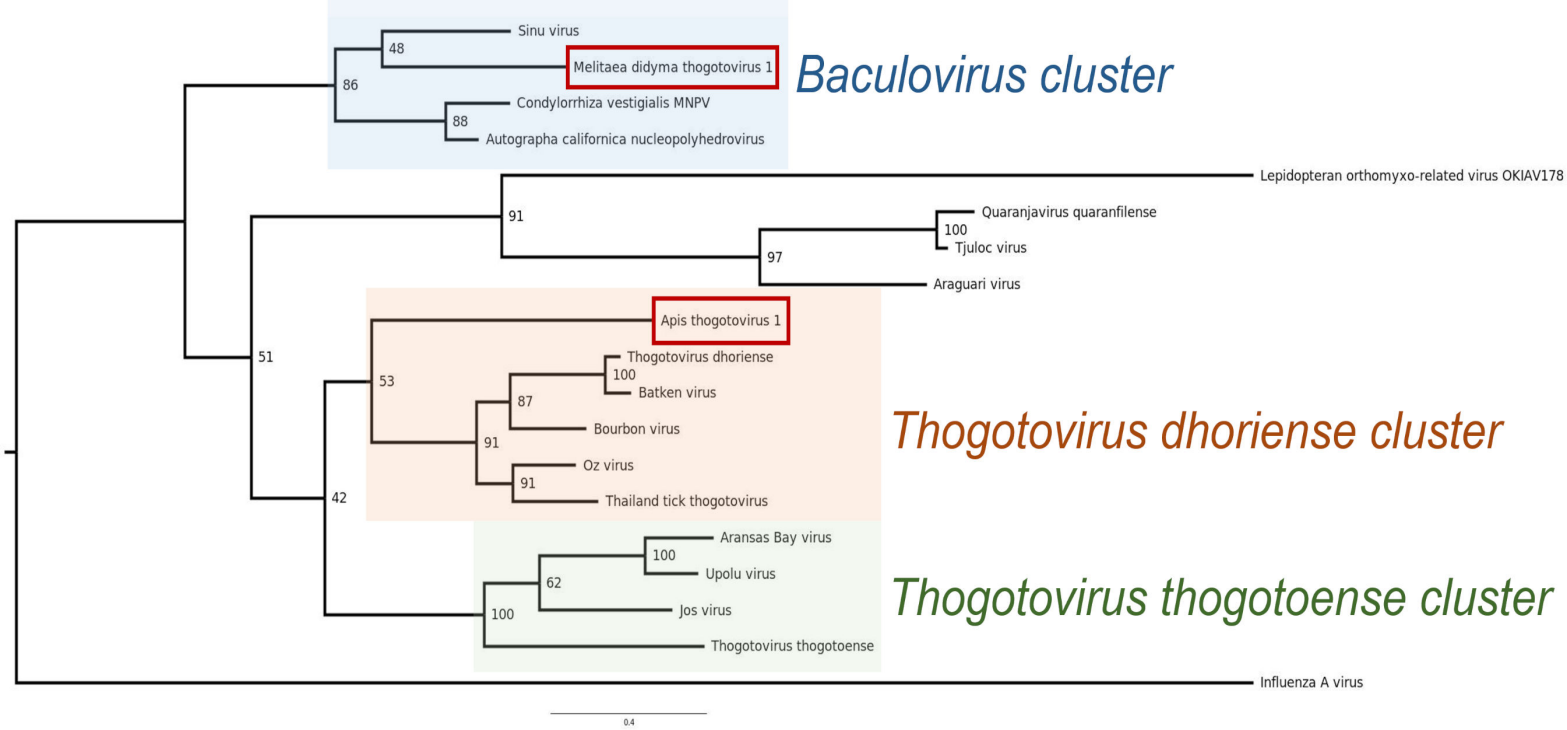
Maximum likelihood phylogeny based on amino acid sequence of GP of Melitaea didyma thogotovirus 1, Apis thogotovirus 1 and viral members of the *Orthomyxoviridae* and *Baculoviridae* families.

In phylogenetic analyses of the proteins encoded by segments among members of the *Orthomyxoviridae* family, all proteins of MediTHOV-1 cluster with Sinu virus proteins (Fig. S2A through E). For the GP protein phylogeny (Fig. 1), sequences of AcMNPV and Condylorrhiza vestigialis MNPV (CoveMNPV) were included. In this latter phylogeny, the GP protein of MediTHOV-1 groups with Sinu virus, albeit with low bootstrap values (Fig. 1). This clade clusters closely with G1-α baculoviruses, in a separated clade from that of other thogotoviruses, indicating their close phylogenetic relationship.

There is currently one known near-complete orthomyxovirus genome from Lepidoptera, Lepidopteran orthomyxo-related virus OKIAV178 (32). Nevertheless, phylogenetic analysis shows that this virus is evolutionary distant from the *Thogotovirus* genus.

### Phylodynamic analysis

**Fig 2:**
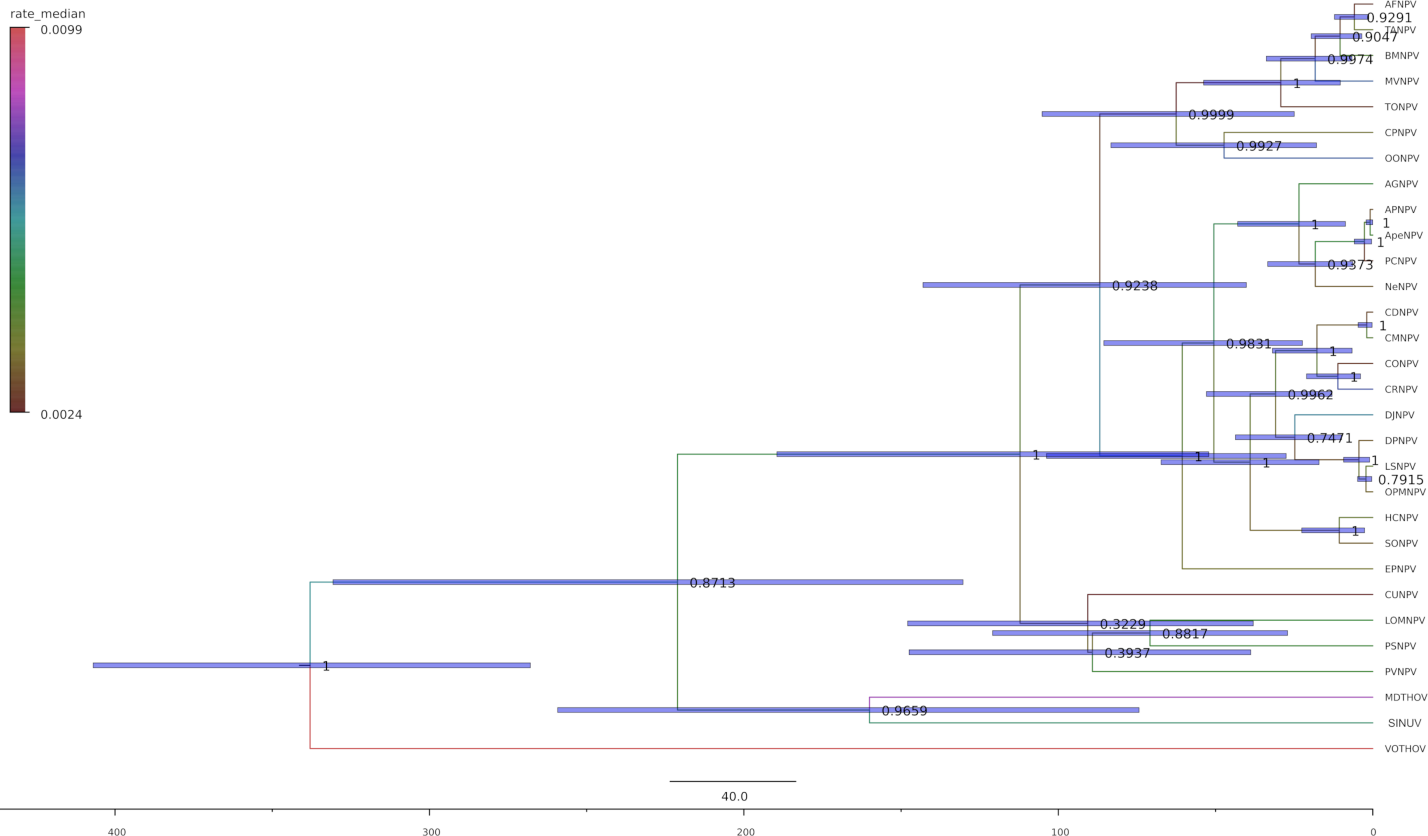
Temporal dynamics of glycoprotein evolution between thogotoviruses and alphabaculoviruses. Node bars depict the 95% HPD interval for age, node labels depict posterior probabilities, and the time axis is in millions of years ago (mya). Branch labels are colored by substitution rate median values.

In order to perform phylodynamic analysis of the shared history of alphabaculovirus and thogotovirus glycoproteins, we analyzed a data set comprising 30 glycoproteins, 27 from baculovirus species and the three most closely related thogotovirus glycoproteins, namely those of Apis thogotovirus 1, Melitaea didyma thogotovirus 1, and Sinu virus (Table S1). Moreover, we assumed a virus co-diverged with their hosts over geological time scales, a pattern commonly observed in the macroevolution of invertebrate virus macroevolution (33), documented for baculoviruses (34), and proposed as a working hypothesis for the order Articulavirales (35). Thus, we used a single calibration point corresponding to the estimated origin time of the Holometabola superorder, approximately 345 million years ago (mya) (36). For the resulting MCC tree, the 95% highest posterior densities (HPD) interval for the node height separating the alphabaculovirus and thogotovirus glycoproteins is 330–130 mya (Fig. 2).

Thezé et al. found a 95% HPD of 189–10 mya for the divergence between alphabaculovirus and betabaculovirus (37). Therefore, there is an overlap of HPDs for the origin of alphabaculoviruses and the acquisition of *gp64* of roughly 60 million years, which spans the late Jurassic to the early Cretaceous periods. This indicates a relatively early acquisition of the glycoprotein by the alphabaculoviruses that also happened during a time of radiation within holometabola, which in turn coincides with flowering plant diversification (36, 37).

### Recombinant virus constructs

**Fig 3:**
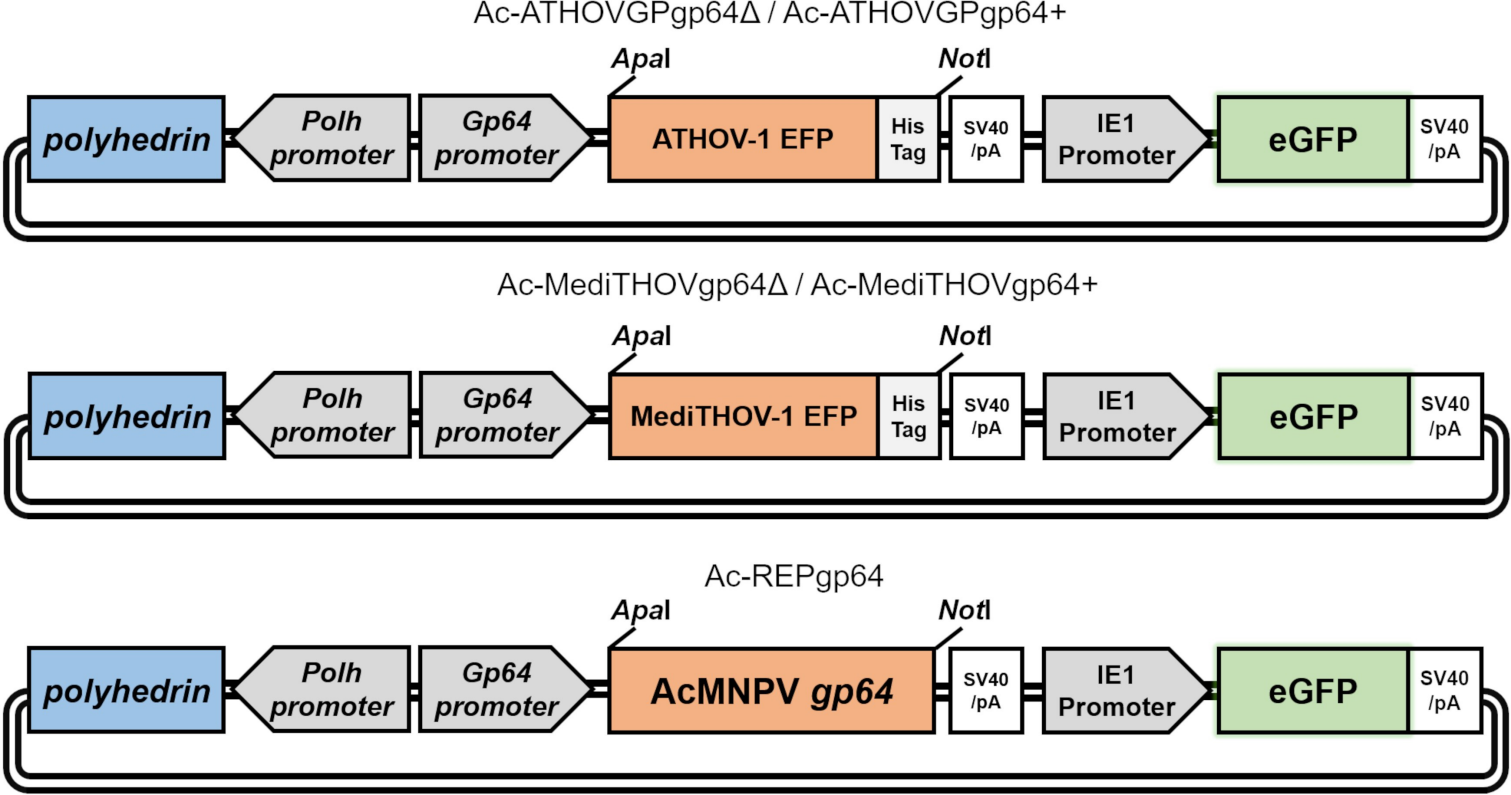
Schematic representation of bacmids Ac-ATHOV-1, Ac-MediTHOV-1, and Ac-REPgp64, constructed carrying heterologous EFP sequences from thogotoviruses, and of control bacmid carrying its native *gp64* gene. The scheme also shows representation of early and late infection reporter genes, eGFP and *polyhedrin*, respectively, as well as regulatory regions and restriction sites utilized for molecular cloning.

The GP64 envelope fusion protein is essential for the entry of its budded virus from G1-α baculoviruses into new cells and for the efficient budding from infected cells (8). We evaluated the ability of genes encoding for the EFP (fused with a C-terminal His-tag) of two thogotoviruses, one novel virus with a lepidopteran host and one with a hymenopteran host, to rescue the infectivity of an AcMNPV deleted for its native *gp64* gene. A bacmid named Ac-REPgp64, repaired for its native *gp64* gene, was used as infection control. Lepidopteran Sf21 cells were transfected with recombinant bacmids Ac-ATHOV-1 and Ac-MediTHOV-1 (Fig. 3), and the cell culture supernatant was passaged in Sf21 cells to evaluate the production of infectious BVs.

### MediTHOV EFP infectivity rescue assays

**Fig 4:**
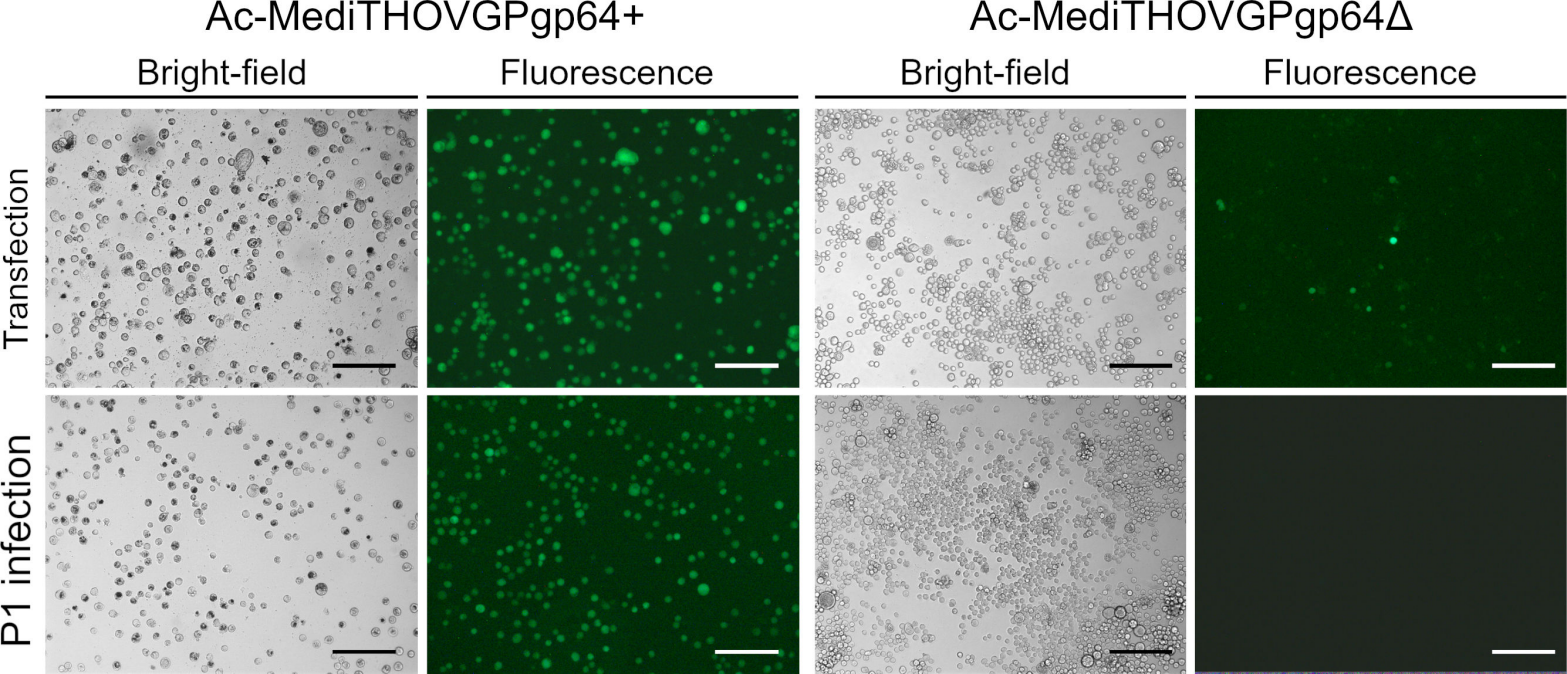
Fluorescence microscopy of Sf21 cells 7 days post-transfection (dpt) and 72 hours post-infection (hpi) of the first serial infection passage (P1) of Ac-MediTHOVgp64+ and 7 dpi of the virus Ac-MediTHOVgp64Δ. Scale bar is 250 µm.

Interestingly, despite the higher percentage of amino acid identity shared between MediTHOV EFP and baculovirus GP64, when compared to other thogotoviruses, this glycoprotein was unable to rescue AcMNPV *gp64 null* infectivity. The cell culture supernatant from Ac-MediTHOVgp64Δ transfection did not produce subsequent infection in either first (P1) or second (P2) passage in Sf21 cells (Fig. 4).

### ATHOV-1 EFP infectivity rescue assays

**Fig 5:**
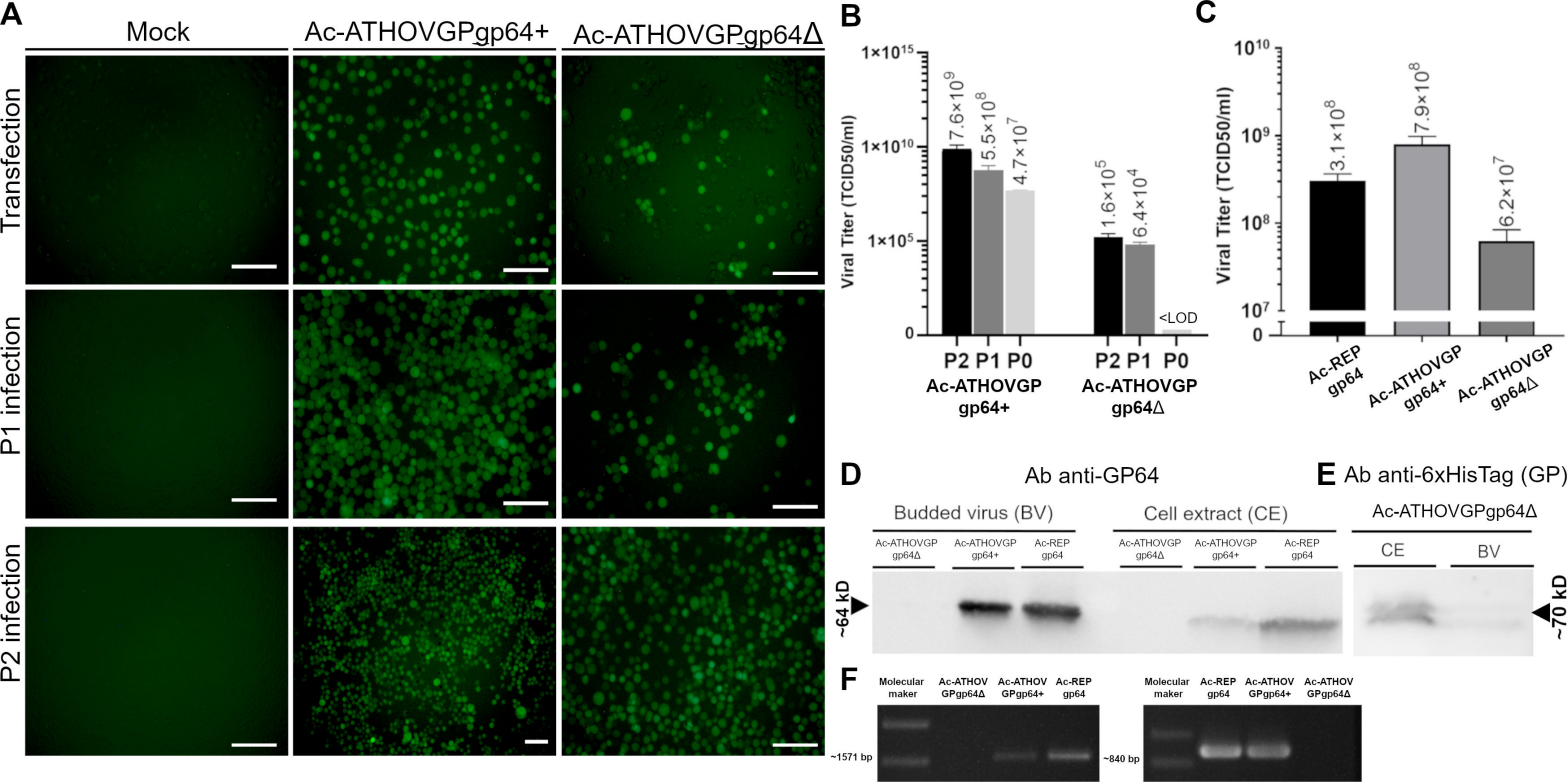
(**A**) Serial infection showing that the envelope fusion protein of ATHOV-1 is able to rescue *gp64* null AcMNPV. Fluorescence microscopy of Sf21 cells 7 dpt, at 72 hpi of two serial infection passages (P1 and P2) of Ac-ATHOVGPgp64+, and at 7 dpi of the virus Ac-ATHOVGPgp64Δ. Scale bar: 125 µm. (**B**) Titer obtained from transfection (P0) at 7 days post-transfection (dpt) and from serial passages (P1 and P2) at 72 hours post-infection (hpi) for AcATHOVgp64+and 7 days post-infection (dpi) for AcATHOVgp64Δ. LOD: limit of detection. (**C**) Titers obtained from infected Sf21 cells of AcATHOVgp64+ and Ac-REPgp64 at 72 hpi and AcATHOVgp64Δ at 14 dpi. (**D**) Western blot of the extract of infected Tn5B cells and budded viruses concentrated from the culture supernatant (BV) labeled with anti-GP64 antibodies and (**E**) antibodies against His-tagged ATHOV EFP. (**F**) PCR confirmation of the deletion of the *gp64* gene, using two *gp64* specific primer pairs, of recombinant virus stocks.

In contrast to the inability of MediTHOV EFP to functionally replace baculovirus native GP64, ATHOV-1 EFP could rescue the infectivity of AcMNPV deleted for its native *gp64* gene. The transfection of recombinant bacmid from Ac-ATHOVGPgp64Δ carrying ATHOV-1 EFP in the absence of native GP64 produced infectious virus particles that were able to produce infection and be serially passaged in Sf21 cells (Fig. 5A).

Lower viral titers were obtained from Ac-ATHOVGPgp64Δ serial infections, averaging around 1 × 10^6^ TCID50/mL, compared to the viruses containing *gp64*, AcTHOVGPgp64+ and Ac-REPgp64, which had titers greater than 1 × 10^8^ TCID50/mL (Fig. 5B). The quantity of infectious viral particles obtained at 7 days post-transfection (P0) was below the limit of detection (LOD) of titration by the end-point dilution method. However, subsequent passages in Sf21 cells led to a considerable increase in viral titer in passages one (P1) and two (P2) (Fig. 5B).

It was consistently observed that higher titers of AcATHOVgp64+ compared to Ac-REPgp64 were obtained, indicating the possibility that the incorporation of the ATHOV-1 EFP in addition to native GP64 acts in a synergistic manner, increasing the efficiency of infectious BV particle production *in vitro* (Fig. 5C). Western blot using an antibody against the histidine tail fused to ATHOV-1 EFP detected the presence of a protein with a molecular mass of the expected size in the extract of infected cells and in concentrated viral particles from the culture supernatant, indicating correct expression and incorporation into the BV (Fig. 5D and E). Immunolabeling using an antibody against the GP64 protein confirmed its absence both in the extract of cells infected with the Ac-ATHOVGPgp64Δ virus and in the concentrated culture supernatant (BV) (Fig. 5D), while PCR confirmed the absence of gp64 in deleted virus stock (Fig. 5F). In contrast, the presence of ATHOV-1 EFP was confirmed in recombinant viruses containing the gene, although at lower concentrations (Fig. 5E).

### Ac-ATHOVGPgp64Δ *in vitro* growth kinetics

**Fig 6:**
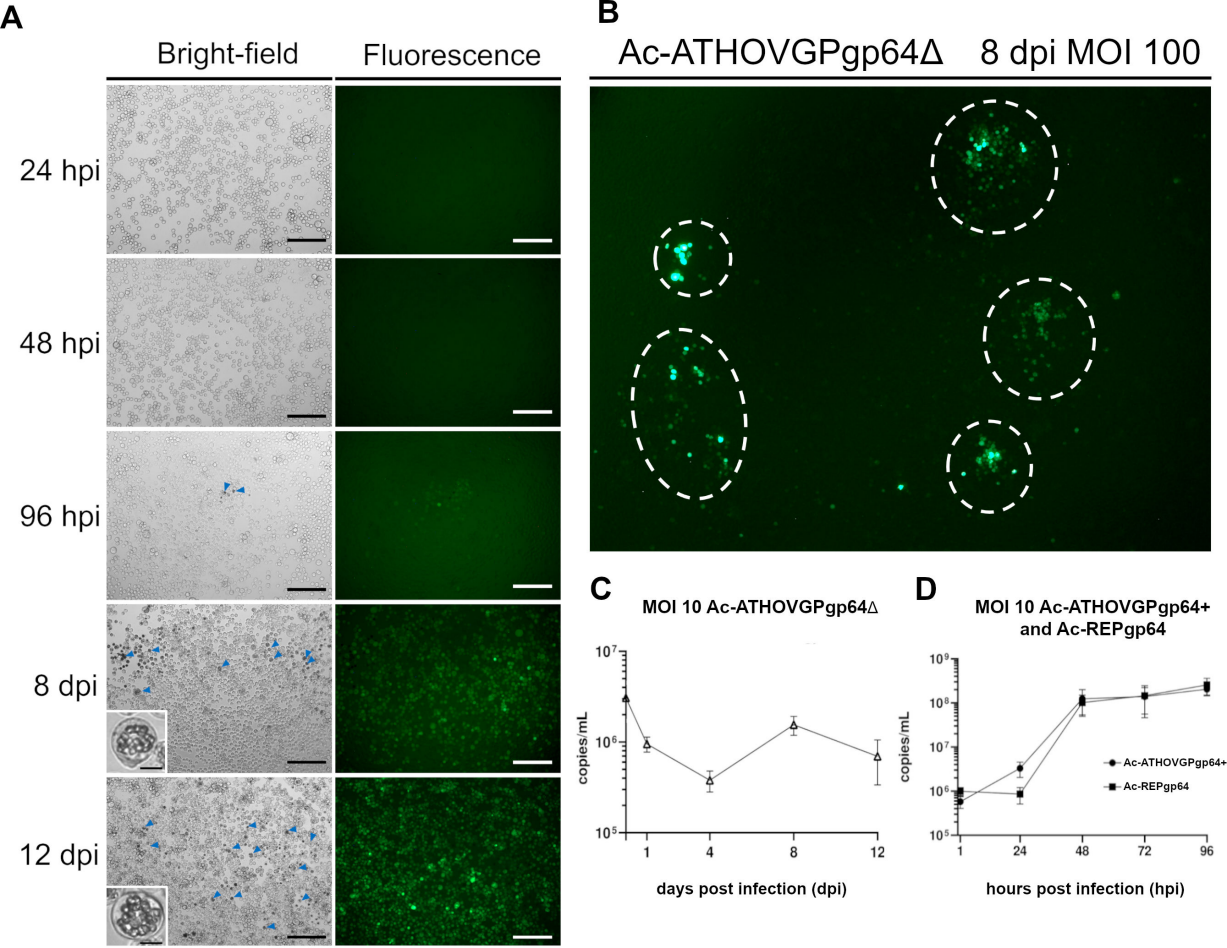
(**A**) Fluorescence and bright-field microscopy of Ac-ATHOVGPgp64Δ infected Sf21 cells, expressing the reporter gene eGFP and polyhedrin (dark intracellular masses, blue arrows, and insets), indicating the progression of infection over time. (**B**) Infected Sf21, dotted circles demarcate regions of isolated infection foci still discernible at 8 dpi. Scale bar: 250 µm and 15 µm. (**C**) qPCR growth curve of Ac-ATHOVGPgp64Δ and of (**D**) GP64 carrying viruses.

The virus Ac-ATHOVGPgp64Δ exhibited delayed replication kinetics compared to AcMNPV carrying GP64 (Fig. 6). Isolated foci of infection could be observed up to 8 dpi (Fig. 6B), indicating ATHOV-1 EFP ability to induce cell-to-cell spread of budded viruses, albeit with lower efficiency than viruses carrying GP64. This observation indicates that viral spread in culture may be more efficient between adjacent cells in close physical proximity.

The qPCR analysis indicates that large quantities of extracellular Ac-ATHOVGPgp64Δ BVs do not accumulate over the infection course (Fig. 6C) when compared with the GP64 carrying viruses (Fig. 6D). The spread of the infection appears to involve a cell-to-cell infection mechanism mediated by ATHOV-1 EFP, corroborating the slow progression pattern and the occurrence of isolated infection foci observed in culture.

This extended infection kinetics *in vitro* was observed in all infections conducted in this study, with maximum spread in culture occurring on average between 12 and 14 dpi. Comparatively, *in vitro* infections by the wild-type AcMNPV (and by recombinants containing the *gp64* gene) show maximum infection spread at 24 hpi.

### ATHOV-1 EFP is able to induce membrane fusion

**Fig 7:**
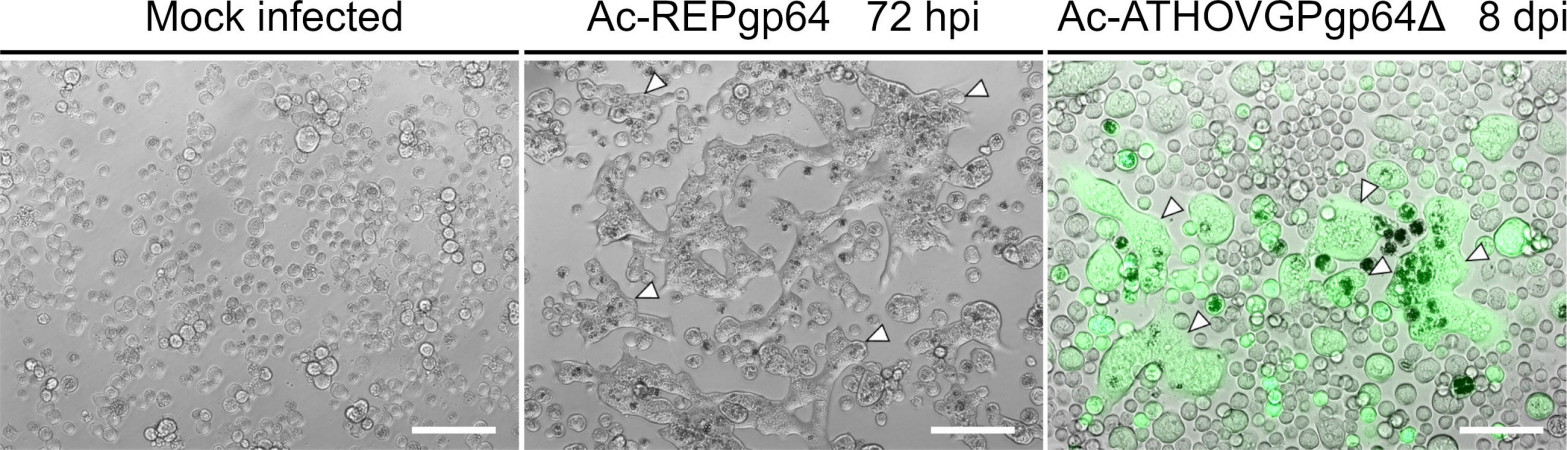
Sf21 cells infected with the control virus Ac-REPgp64 and the virus Ac-ATHOVGPgp64Δ after exposure to low pH. Syncytia are indicated by white arrowheads. Overlay of fluorescence and bright field demonstrates syncytia composed of cells expressing the reporter gene eGFP. Scale bar: 125 µm.

During natural baculovirus infection, AcMNPV GP64 undergoes conformational changes in response to the acidification of late endosomes, catalyzing membrane fusion and virus release in the cytoplasm (23, 38). Cells infected with Ac-ATHOVgp64Δ and subjected to acidified culture medium exhibited the formation of large syncytia, indicating a similar mechanism of action as GP64 of the ATHOV-1 EFP in the context of baculovirus infection (Fig. 7).

Induced syncytium formation only occurred after sufficient infection spread at 8 dpi, possibly due to the need for close physical contact between infected cells expressing ATHOV-1 EFP in sufficient quantities to mediate fusion between adjacent cell membranes.

### Enhanced transduction in mosquito cells

**Fig 8:**
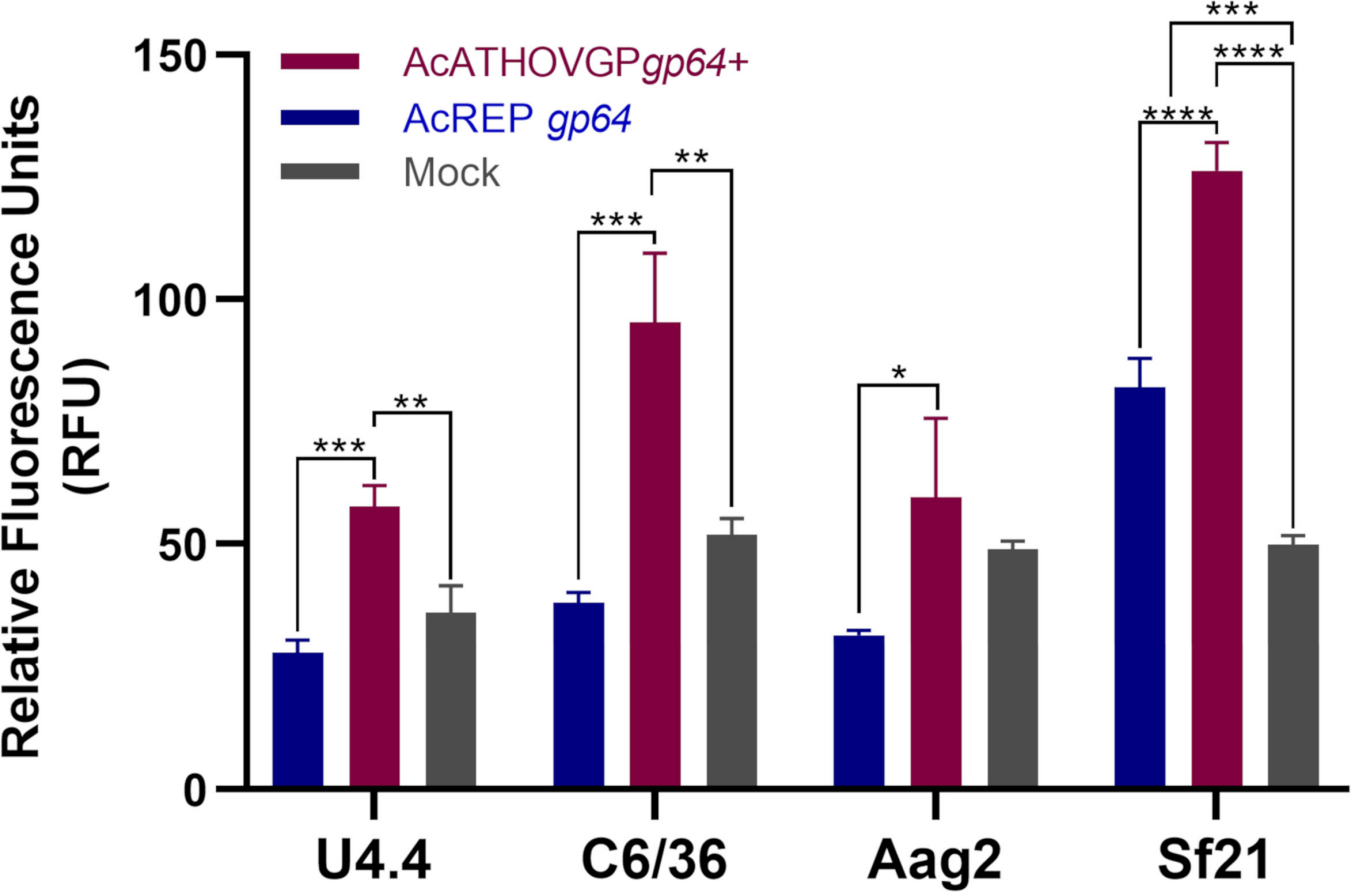
ATHOV-1 EFP enhances transduction efficiency in mosquito cells U4.4 and C6/36, and lepidopteran cell line Sf21. Total fluorescence emitted by the eGFP reporter protein from lysed cells measured at 48 hpi at MOI 5. Significance performed by Tukey post-test: *P < 0.03, **P < 0.002, ***P < 0.0005, and ****P < 0.0001.

The capability of AcMNPV to enter various cell lines and transiently transduce genes enables its use for gene delivery and protein production in a wide range of cell lines (39). We evaluated the capacity of ATHOV-1 EFP to enhance AcMNPV entry into the mosquito cell lines C6/36, U4.4, and Aag2, which are non-permissive to its replication, as well as into permissive Sf21 lepidopteran cell line. To assess entry and transduction capacity in these cell lines, we evaluated eGFP expression under the control of the AcMNPV *ie-1* early gene promoter, which is functional in mosquito cells (40). Interestingly, the Ac-ATHOVGPgp64+ virus showed higher levels of eGFP expression in most tested cell lines, as observed by fluorescence microscopy (Fig. S3). This suggests that the incorporation of the ATHOV-1 envelope fusion protein (EFP) enhances AcMNPV transduction efficiency. Quantification by fluorimetry confirmed significantly increased eGFP levels in C6/36, U4.4, and Sf21 cells when compared to Ac-REPgp64. In Aag2 cells, however, no statistically significant difference was observed despite a visually stronger fluorescence signal. This discrepancy between qualitative (microscopy) and quantitative (fluorometry) data may reflect differences in cellular autofluorescence or protein expression thresholds across assays (Fig. 8).

### Cryo-EM shows differential incorporation of EFPs in BV envelope

**Fig 9:**
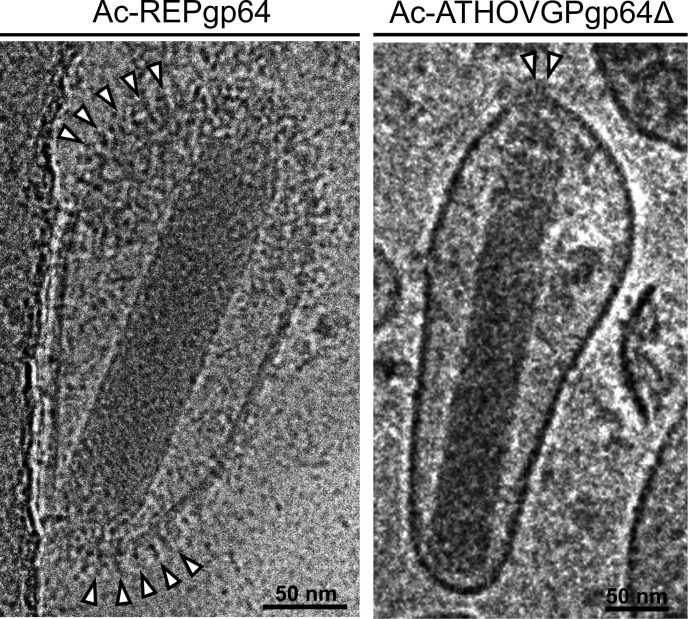
Cryo-EM comparison of AcMNPV *gp64* null rescued with its native *gp64* gene (Ac-REPgp64) and with heterologous ATHOV-1 EFP (Ac-ATHOVgp64Δ). White arrowheads indicate visible peplomers composed of envelope glycoproteins.

Cryo-EM analysis of BVs indicates a significantly lower level of incorporation of the heterologous ATHOV-1 EFP in viral envelopes, when compared to the wild-type GP64 (Fig. 9). Ac-REPgp64 BVs displayed many copies of protruding envelope peplomers, while viral particles of Ac-ATHOVGPgp64Δ showed very few copies of such structures composed of ATHOV-1 EFP, or no easily discernible spikes at all (Fig. 9; Fig. S3). When present, these structures localized in the extremities of BVs similarly to the localization of GP64 (40).

This observation corroborates the viral growth kinetic data and suggests that the critical factor for the capacity of heterologous EFPs to rescue the infectivity of *gp64* null baculovirus may be EFP envelope incorporation. This process could recapitulate evolutionary events necessary for the acquisition of *gp64* by the ancestor G1-α baculoviruses.

## DISCUSSION

All known alphabaculoviruses infect Lepidoptera (9). Therefore, we have searched the Serratus platform for RdRp sequences similar to those of Sinu virus in lepidopteran transcriptome data. This was motivated by the clustering of the Sinu virus glycoprotein with GP64 sequences of baculoviruses in phylogenetic trees. Through this approach, we discovered a novel thogotovirus in the *Melitaea didyma* butterfly, which we have named Melitaea didyma thogotovirus 1 (MediTHOV-1). This study demonstrates that data mining, combined with inferred HGT events, can be used in a hypothesis-driven manner to discover novel viruses, emphasizing that HTS data should be utilized not only for the discovery of new viruses but also to infer the evolutionary and physiological contexts in which viruses play significant roles (41).

The identification of MediTHOV-1, whose glycoprotein groups closely with baculovirus GP64 and Sinu virus sequences, supports the hypothesis that this baculoviral protein originated from an HGT event involving an ancestral orthomyxovirus. However, the exact mechanism of how a co-infection event might have occurred during the same host developmental stage remains unclear.

Given the phylodynamics, we believe that the *gp64* gene gain early in the evolution of alphabaculoviruses contributed to its dispersal among various lepidopteran species first, and then these viruses have co-evolved with their hosts to the present day, providing negative selection pressures on *gp64*. Indeed, present-day alphabaculovirus genomes have the lowest non-synonymous to synonymous (Ka/Ks) substitution rates in their *gp64* genes (42). This would explain why this group of viruses is the most diverse within *Baculoviridae* despite most viruses in the genera being species-specific for their hosts. However, we cannot exclude co-occurring hypotheses for the observed higher species diversity of viruses in *Alphabaculovirus* compared to the rest of the family. Namely, alphabaculoviruses and betabaculoviruses have been used for the biological control of agronomic pests (9), thus motivating the discovery of new viruses against new pests. In addition, it is possible that Apis thogotovirus 1 ancestors originated from mites and spilled from them into bees at later geological times than our assumed calibration point. Overall, we find the first hypothesis to be more likely, at least when comparing the diversity of G1-α and G2-α viruses.

Although baculoviruses primarily infect insects at the larval stage, they have also been observed in adult individuals in a covert form, characterized by the absence of visible signs of infection (43). This covert infection can result from sublethal infections during larval development and can also enable vertical transmission (44). Such occurrences could facilitate a coinfection event between an ancestral baculovirus and thogotovirus within the same adult individual. Additionally, it is possible that orthomyxoviruses evolutionarily close to MediTHOV-1, which infect larvae, exist.

While baculoviruses have been pseudotyped in numerous studies (26, 39, 45, 46), there are fewer studies on pseudotyping of thogotovirus glycoproteins (47). Lung et al. (26) were able to successfully pseudotype AcMNPV with baculovirus F and VSV F proteins, indicating that pseudotyping is also possible with EFP of unrelated viruses, while evaluating functional compatibility. However, the authors report that Thogoto virus EFP was unable to rescue the infectivity of pseudotyped AcMNPV in insect cell lines (26). Therefore, to the best of our knowledge, this study represents the first description of a successful description of a thogotovirus EFP pseudotyping in a baculovirus that partially replaced native GP64 activity.

The glycoprotein of certain members of the *Thogotovirus* genus, such as those in the Dhori group, allows these viruses to infect both invertebrates and vertebrates (48). Similarly, the GP64 of G1-ɑ baculoviruses enables their use as vectors for gene therapy and other biotechnological applications in mammalian and mosquito cell lines (39, 49).

The insertion of *gp64* within ancestors of G1-ɑ may have been a determining event for the formation of G1-ɑ in a different lineage from G2-ɑ, as it was found to be the protein with the least amino acid changes among the 11 genes present exclusively in G1-ɑ (41). Given its stability over evolutionary time, it is not surprising to encounter difficulties in pseudotyping this protein for orthologs. While it is possible to pseudotype G2-α with GP64 in place of its F protein (44), up until recently, the pseudotyping of thogotovirus glycoproteins in place of GP64 has only been successful as an enhancer of budded virus transduction in mammalian cells (45).

Here, we describe the successful pseudotyping of a *gp64* null AcMNPV with a bee thogotovirus envelope glycoprotein, resulting in the recombinant baculovirus regaining infectivity in lepidopteran cell lines, albeit less efficiently than the wild-type virus infection. In contrast, pseudotyping with MediTHOV-1 glycoprotein did not rescue AcMNPV *gp64* null infectivity. It has been shown that the domain II and the stalk portion of the domain III baculoviral and thogotoviral proteins changed the most in respect to their electrostatic surface, which could correspond to host-specific tropism (25, 50). Aligned to this notion, we have observed that MediTHOV-1 domains were more positively charged than ATHOV-1, which seemingly relates to the tropism of the former virus to lepidoptera, in similarity to AcMNPV (Fig. S4). In spite of this, the MediTHOV-1 protein failed to rescue its function of AcMNPV.

We believe that certain sequence features in the MediTHOV-1 EFP contribute to the failure in rescuing infectivity. The predicted encoded EFP of this virus does not have an obvious signal peptide (Fig. S5). In addition, Yu and colleagues (51) demonstrated that the 388Y residue in the domain IV of GP64 is necessary for generating viral titers of AcMNPV in the Sf9 cell line (50). While all baculovirus sequences analyzed in their study have this residue, some thogotoviruses, including MediTHOV-1, present a 388F residue, while ATHOV-1 carries the 388Y residue (Fig. S6). The complex phylogenetic relationships among lepidopterans, such as *Autographa californica* in Noctuidae and *Melitaea dydima* in Nymphalidae families, may guide divergent evolutionary paths for their viruses over time.

While ATHOV-1 fusion protein restored infectivity, the infection occurred in localized foci within the cell cultures. The resulting BVs displayed fewer GPs in their surface compared with the wild-type virus, as observed through cryo-EM. The number of EFPs in close physical proximity may play a crucial role in overcoming the energetic barrier for hemifusion between viral and cell membranes, as seen with some class I and class II fusion proteins (52). This could explain the localized infection *foci* and lack of extensive spread in culture observed during the infection by Ac-ATHOVgp64Δ*,* which appears to incorporate very few copies of ATHOV-1's EFPs. Consequently, cell-to-cell contact may be required to achieve a sufficient concentration of EFPs to mediate viral spread to adjacent cells.

Hodgson et al. (52) identified various host proteins involved in GP64 trafficking to the plasma membrane, such as Rabs and adaptor proteins associated with endosomal trafficking (52). We find it plausible that, as ATHOV-1 evolved to infect bees and mites, its glycoprotein may have become poorly adapted to interact with the endosomal machinery of lepidopteran cells. This maladaptation could result in suboptimal trafficking from the endoplasmic reticulum to the plasma membrane and/or enhanced endocytosis of the proteins back into the cell. The transmembrane domain of AcMNPV GP64 is important for complete membrane fusion and pore formation, as well as for sub-cellular transport and virus budding (53). In a substitution assay, replacing the transmembrane domain of AcMNPV GP64 with that of THOV generated infectious BVs and promoted full membrane fusion (53).

We also believe that a minimum compatibility between thogotovirus matrix proteins and their counterparts in baculovirus was necessary for the interchangeability of the envelope glycoprotein between these groups. The deletion of the baculoviral *me53* in AcMNPV results in up to a 1,000-fold reduction of BV yield and is important for virion production (54, 55). During late phase infection, Me53 localizes on the plasma membrane of host cells and requires previously anchored GP64 in the membrane to form budding sites for the virus (56). Me53 in the budding sites may function analogously to the matrix protein of Influenza viruses, members of the *Orthomyxoviridae* family, which recruits other viral proteins to facilitate viral budding (56). In this manner, compatibility between interacting domains of Me53 and acquired EFP could be necessary for its efficient incorporation in the viral G1-α envelope. However, further experiments are needed to unravel the interaction between thogotovirus EFP with baculovirus and lepidopteran cells.

Our study was able to replicate several observations made by Wang and colleagues (57) in their cryo-EM study of baculoviral budded virus morphology (57). These included virions with stretched sides, the presence of vesicles within the virions, BVs with multiple capsids, and relaxed forms of capsids (Fig. S7) despite using centrifugation prior to cryo-EM to enrich for BVs in our samples.

The incorporation of thogotovirus GPs in the BV envelope has been explored as a strategy to enhance its capacity to enter and transduce genes in mammalian cells (46). Surprisingly, the recombinant baculovirus carrying both its native GP64 and the EFP of ATHOV-1 demonstrated a greatly enhanced ability to transduce reporter genes in mosquito cell lines, indicating its potential as a tool for gene delivery and heterologous protein expression in these cell lineages. Additionally, the increased transduction in the permissive Sf21 lepidopteran cell line suggests a synergistic effect of ATHOV-1 EFP in conjunction with AcMNPV GP64 in enhancing *in vitro* infection. This synergistic effect could recapitulate the evolutionary steps that led to the substitution of F and the main baculovirus fusion protein by GP64 in G1-α (58). These findings further suggest potential applications of this viral vector for heterologous protein expression and gene delivery, which are currently under study by our group.

## MATERIALS AND METHODS

### Bioinformatic analyses

The Sequence Read Archive was explored using the Serratus platform (31) (https://serratus.io/explorer/rdrp; accessed on 12 December 2022) to search for data sets containing reads with at least 45% identity with the three catalytic motifs of the RNA-dependent RNA polymerase of Sinu virus, the thogotovirus whose glycoprotein is most similar to those of baculoviruses (25). We identified an RNA-seq data set (59) (SRR1325001) of the spotted fritillary butterfly (*Melitaea didyma*) containing reads with 45% similarity to the conserved domains of RdRp of Sinu virus. After downloading the RNA-seq data set (59), reads were assembled into transcripts using RNAspades v3.15.5 (60). We then performed a BLASTx of the transcripts against a local database containing all thogotovirus proteins from the NCBI protein database (downloaded on 11 October 2022). We identified six nucleotide sequences corresponding to the thogotovirus segments, which were annotated using NCBI ORFfinder (https://www.ncbi.nlm.nih.gov/orffinder; accessed on 15 December 2022). Nucleotide sequence data reported are available in the Third Party Annotation (TPA) section of the DDBJ/ENA/GenBank databases under the accession numbers BK068795-BK068800.

Maximum likelihood phylogenetic trees of each segment were constructed with the protein sequences, trimmed with trimAl v1.4.rev15 (61). The chosen substitution model was selected with modeltest-ng v0.1.7 (53), and the tree was constructed with RaxML-NG v1.0.3 (62) with 1,000 bootstraps and visualized with figtree (http://tree.bio.ed.ac.uk/software/figtree).

Signal peptides for AcMNPV, ATHOV-1, and MediTHOV-1 were predicted using signalP 6.0 (https://services.healthtech.dtu.dk/services/SignalP-6.0) (63). Multiple sequence alignment between baculovirus and thogotovirus glycoproteins was generated with MAFFT v7.489, and the resulting alignment was highlighted by hydropathy using Texshade (64, 65).

### Phylodynamic analysis

We generated a time-calibrated phylogeny of Baculovirus and Thogotovirus glycoproteins using BEAST v2.7.7 (66). Accession numbers for the sequences are available in Table S1. We used a Yule Speciation model and a normally distributed calibration point at the root of the tree, based on the origin of Holometabola described in Misof et al. (36) (mean of 345 million years ago; sigma of 35 mya) (36). In addition, we have used the LG substitution model, a relaxed log-normal clock, and an initial Unweighted Pair Group Method with Arithmetic Mean (UPGMA) tree, chosen using BEAUti v2.7.7 (67). The resulting model was run with a Markov Chain Monte Carlo (MCMC) with 10 million states. Conversion and Effective Sample Size (ESS), which was accepted over 200 for the parameters, were evaluated using Tracer v1.7.2 (68). A maximum Clade Credibility tree (MCC) was constructed with TreeAnnotator after a 10% Burn-in (https://www.beast2.org/treeannotator/) and rendered using Figtree (http://tree.bio.ed.ac.uk/software/figtree/).

### Cell culture and viruses

All insect cell lines were maintained at 27°C in TC-100 culture medium (Vitrocell Embriolife) supplemented with 10% fetal bovine serum (FBS) (Gibco). Sf21 cells (69) derived from the lepidopteran *Spodoptera frugiperda* were used for all virus amplification, transfection, and infection assays, while *Trichoplusia ni* Tn5B cells (70) were used for protein expression assays. Virus titration was carried out using Sf-9 Easy Titer (Sf-9ET) cells (71). *Aedes aegypti* derived Aag2 mosquito cells and *Aedes albopictus* derived C6/36 (72), U4.4 (73), were used for transduction assays. Recombinant baculoviruses were generated utilizing the Bac-to-Bac baculovirus expression system (Thermo Fisher Scientific) according to the manufacturers' instructions.

### Molecular cloning and recombinant baculovirus construction

The coding sequence for the Apis thogotovirus 1 (ATHOV-1) and that of Melitaea didyma thogotovirus 1 (MediTHOV-1) envelope glycoproteins were chemically synthesized into gBlocks gene fragments by Integrated DNA Technologies (IDT). The sequences were amplified by PCR using the primer pairs listed in Table S2. A histidine tag sequence was added to the 3′ terminus of the glycoprotein genes for immunodetection of protein expression. The amplified sequences were digested with the enzymes *Apa*I and *Not*I and subsequently ligated into the previously digested donor plasmid pFB-Acgp64-pA-PG (45), under the control of the baculovirus *gp64* promoter. This plasmid also contains the eGFP reporter gene and the polyhedrin gene under the control of the IE1 and polyhedrin baculovirus promoters, respectively. The resulting plasmids were named pFB-ATHOVGP and pFB-MediTHOV (Fig. 1), and their correct sequences were confirmed by restriction enzyme digestion and by Sanger sequencing.

Plasmids pFB-ATHOVGP and pFB-MediTHOV were used to generate recombinant baculoviruses carrying either ATHOV-1 or MediTHOV-1 EFP genes, but lacking the baculoviral *gp64* gene (named Ac-ATHOVGPgp64Δ and Ac-MediTHOVgp64Δ), through Bac-to-Bac (Thermo Fisher Scientific) transposition into a *gp64* null bacmid (26). Additionally, recombinant baculoviruses carrying both the Thogotovirus EFP and the *gp64* gene (named Ac-ATHOVGPgp64+ and Ac-MediTHOVgp64+) were also constructed using the Bac-to-Bac expression system by transposition into a bacmid possessing the native baculovirus *gp64* gene. Finally, a recombinant baculovirus with the wild-type AcMNPV *gp64* gene also inserted under the control of the *gp64* promoter and transposed into a *gp64 null* bacmid (named Ac-REPgp64) was used for comparison in infection and replication assays (44) (Fig. 2).

### Transfection and infection assays

A total of 1.3 µg of recombinant bacmid DNA from Ac-ATHOVGPgp64Δ, AcTHOVGPgp64+, Ac-MediTHOVgp64+, Ac-MediTHOVgp64Δ, and Ac-REPgp64 DNA was transfected in duplicate into 8 × 10^5^ Sf21 cells in six-well plates (Cellstar; Greiner BioOne) using the FuGENE HD transfection Reagent (Promega) according to manufacturer's specifications. Transfected cells were monitored daily for the appearance of fluorescence from the early infection reporter eGFP gene and for polyhedra formation as a reporter for late phase infection. Culture supernatant from the transfection of insect cells (P0) was collected 7 days post-transfection (dpt) and clarified by centrifugation at 5,000 × *g* for 6 min. Subsequently, 200 µL of P0 inoculum was used to infect 8 × 10^5^ Sf21 cells in six-well plates (Cellstar; Greiner Bio-One). This first passage was monitored for signs of infection and formation of infection foci.

In subsequent passages, Ac-ATHOVGPgp64Δ and Ac-MediTHOVgp64+ infected culture supernatant (P1) was collected and clarified 7 days post-infection (dpi), while for Ac-ATHOVGPgp64+, Ac-MediTHOVgp64+, and Ac-REPgp64, supernatants were collected at 72 hours post-infection (hpi). These supernatants were then passaged in Sf21 cells to observe the propagation of infection and collection of cell culture supernatant (P2). All passage supernatants were titrated in triplicates using Sf9 Easy Titer (ET) cells by endpoint dilution to establish the 50% tissue culture infective dose (TCID_50_) (68).

### Analysis of protein expression

The expression of the recombinant Thogotovirus membrane EFP was analyzed using sodium dodecyl sulphate polyacrylamide gel electrophoresis (SDS-PAGE) followed by Coomassie Blue Staining and by Western blotting of infected cell extracts, as well as concentrated recombinant virus particles from the culture supernatant. Tn5B cells were infected at a multiplicity of infection (MOI) of 10 in T-75 flasks, and cell cultures were collected at 10 dpi for Ac-ATHOVGPgp64Δ and at 72 hpi for Ac-ATHOVGPgp64+ and Ac-REPgp64. The culture supernatant was clarified by centrifugation at 5,000 × *g* for 6 min, filtered through a 0.22 µm Syringe filter (Millipore), followed by viral particle concentration by ultracentrifugation at 150,000 × *g* for 1 h and 20 min.

For Western blotting, samples were denatured in loading buffer containing β-mercaptoethanol at 95°**C** for 10 min, subjected to SDS-PAGE, and transferred to a PVDF membrane. The membrane was blocked in 3% bovine serum albumin (BSA) in phosphate buffered saline (PBS) for 1 h at room temperature, washed once for 5 min with PBS, and then incubated with either Anti-6× His tag mouse monoclonal antibody (H8, Invitrogen) at a 1:3,000 dilution, or with Anti gp64 monoclonal antibody (AcV5, Invitrogen) at 1:1,000 dilution in PBS for 1 h at room temperature. The membrane was then washed five times for 5 min each with PBS and 0.05% Tween 20 (PBST) and incubated with an alkaline phosphatase conjugated goat anti-mouse IgG (Sigma-Aldrich) at a 1:10,000 dilution for 1 h at room temperature. The membrane was subsequently washed five times for 5 min each with PBST, and proteins were detected using NBT/BCIP substrate (Promega).

### PCR confirmation of *gp64* deletion in recombinant virus

DNA was extracted from 200 µL of second passage virus inoculum from viruses Ac-REPgp64 and AcATHOVGPgp64+ at 72 hpi and for virus Ac-ATHOVGPgp64Δ at 14 dpi, using the QIAamp DNA Mini Kit (Qiagen) according to manufacturer's specifications. Nucleic acids were eluted on 200 µL of buffer AE. One microliter was used in PCR reactions using GoTaq Green Master Mix (Promega) with primers for amplification of the whole *gp64* gene: F-AcMNPVGp64 5-ATAGGGCCCATGGTAAGCGCTATTGTT-3′ and R-AcMNPVGp64 5′-ATAGCGGCCGCTTAGTGATGGTGATGGTGATGATATTGTCTATTACGGTT-3′ (expected band length = 1,571 bp) or with reverse primer R-AcMNPVGp64lap 5′-TGGCCGCTTCTTGACTCG-3′ internal to *gp64* (expected band length 840 bp). Cycling program used was initial denaturation 95°C for 4 min followed by 35 cycles of 95°C for 30 sec, 53°C for 30 sec, and 72°C for 2 min and final extension at 72°C for 7 min. PCR products were separated on a 0.8% agarose gel.

### Low pH-induced fusogenic activity

The ability of the ATHOV-1 envelope glycoprotein to induce cell membrane fusion under low pH conditions was assessed. Sf21 cells were seeded in triplicate into a 12-well plate and infected at a MOI of 10 with recombinant baculoviruses. At 72 hpi for Ac-REPgp64 and mock-infected cells, and at 8 dpi for Ac-ATHOVGPgp64Δ, the cell culture media were replaced with acidified unsupplemented TC-100 media (pH 4.5) and incubated for 10 min. The cells were then washed once with unacidified TC-100 media. After 1 hour, the induction of syncytium formation was observed under bright-field and fluorescence microscopy.

### qPCR analysis of viral growth kinetics

Sf21 cells were infected at MOI 10 in duplicate in 96-well plates at 50% confluence. Culture supernatant was collected at five time points post-infection: 1 hpi, 24 hpi, 96 hpi, 8 dpi, and 12 dpi for Ac-ATHOVGPgp64Δ virus; and 1 hpi, 24 hpi, 48 hpi, 72 hpi, and 96 hpi for Ac-ATHOVgp64+ and Ac-REPgp64 viruses. The total volume of supernatant from each well was clarified by centrifugation at 4,000 × g for 6 minutes, and total DNA was extracted using the QIAmp Viral RNA Kit (Qiagen) following the manufacturer's recommendations. All samples from the same infection collection time for the three viruses were extracted simultaneously and eluted in a final volume of 60 µL of AVE buffer (ultrapure nuclease-free H_2_O with 0.04% sodium azide). A standard curve was established with the plasmid pCR2.1-TOPOAcIE1, with primers AcIE1FWR (5′-CCATCGCCCAGTTCTGCTTA-3′) and AcIEREV (5′-CTGTTCAAGGGTTGCACAGC-3′), targeting AcMNPV *ie1* gene.

The qPCR reactions were performed in duplicate for each sample using LightCycler 480 SYBR Green I Master reagent (Roche) with 1 µL of eluted DNA as the template. The reactions were carried out in the QuantStudio 5 thermocycler (Thermo Fisher Scientific) with the following cycling program: an initial DNA polymerase activation step at 95°C for 5 minutes, followed by 40 cycles of 95°C for 15 seconds, 60°C for 10 seconds, and 72°C for 20 seconds, followed by a melting curve step. SYBR Green fluorescence was read, and the data were analyzed using QuantStudio Design & Analysis Software (version 1.5.1; Thermo Fisher Scientific).

### Cryo-EM analysis

Virus stocks of Ac-ATHOVGPgp64Δ (200 mL), Ac-ATHOVGPgp64+ (50 mL), and Ac-REPgp64 (50 mL) were filtered through 0.22 µm syringe filters (Millipore) and concentrated by ultracentrifugation through a 25% sucrose cushion diluted in PBS 1×, pH 7.4 at 100,000 × g for 1 h and 20 min. Viral pellets were resuspended in 100 µL of PBS and stored at 4°C until use. Cryo-EM grids were prepared by plunge freezing in liquid ethane using the Vitrobot II System (Thermo Fisher Scientific), at 22°C with the sample volume of 3 µL utilizing QUANTIFOIL Holey Carbon Film R2/2 #200 grids. The prepared grids were then analyzed in the Titan Krios G3i (Thermo Fisher Scientific) transmission electron microscope at LNNano – Brazilian Nanotechnology National Laboratory (CNPEM/MCTI).

### Fluorimetry of transduction in mosquito cells

C6/36, U4.4, Aag2, and Sf21 cells were transduced in triplicates in 24-well plates at an MOI of 5 with Ac-ATHOVGPgp64+ and Ac-REPgp64 viruses. Cells were monitored for fluorescence, and at 48 hpi, they were washed once with PBS and then resuspended and lysed in 150 µL of modified RIPA buffer (Tris-HCl, 50 mM; NaCl ,150 mM; EDTA, 5 mM; Triton x-100, 1%) under agitation for 30 min at 4°C. The cellular lysate was clarified by centrifugation at 17,000 × g for 20 minutes at 4°C. The supernatant was collected, and 100 µL was transferred to a black reading plate. Fluorescence was read with an excitation filter at 475 nm (blue) and an emission filter at 500–550 nm using the GloMax Discover equipment (Promega). Data were plotted in GraphPad Prism 8, tested for normality using the Shapiro-Wilk test, and analyzed by one-way ANOVA, followed by Tukey's post hoc test to compare infection groups within each cell line.

### Protein structure prediction and analysis

Protein structures for ATHOV and MediTHOV glycoproteins were predicted using Colabfold v1.5.5 (74) and AcMNPV GP64 in post-fusion conformation (PDB ID: 3DUZ) (75) as a template. Images corresponding to protein structure electrostatic surface and predicted local distance difference test (pLDDT) values were rendered using ChimeraX v1.9 (76).

### Conclusion

We have provided functional evidence supporting an HGT event of the envelope glycoprotein of thogotoviruses to baculoviruses, and we have discovered a novel thogotovirus in lepidoptera, the first found in the same class of hosts as G1-α baculoviruses. These findings open new avenues of research to better understand the biology of thogotoviruses, the adaptation of baculovirus machinery to receiving a new gene from thogotoviruses, and the biotechnological applications of incorporating thogotovirus EFPs in viral vectors.

## Data Availability

The nucleotide sequence of Melitaea didyma thogotovirus 1 (MediTHOV-1) is available in the Third Party Annotation (TPA) section of the DDBJ/ENA/GenBank databases under the accession numbers BK068795-BK068800. All other sequences cited in the work are publicly available in GenBank under the referenced accession numbers.

## SUPPLEMENTAL MATERIAL

Fig. S1 jvi.02148-24-s0001.tif Structural comparison of baculoviral GP64 from AcMNPV and thogotoviral EFP from *Thogotovirus dhoriense*.

Fig. S2 jvi.02148-24-s0002.tif Maximum likelihood phylogenies based on amino acid sequence of proteins from Melitaea didyma thogotovirus 1 and viral members of the Orthomyxoviridae, including ATHOV-1.

Fig. S3 jvi.02148-24-s0003.tif Fluorescence microscopy of transduction in mosquito cell lines.

Fig. S4 jvi.02148-24-s0004.tif Modeled structural comparison of EFPs from ATHOV-1 and MediTHOV-1.

Fig. S5 jvi.02148-24-s0005.tif SignalP prediction of signal peptides between the glycoproteins of AcMNPV, MediTHOV, and ATHOV-1.

Fig. S6 jvi.02148-24-s0006.tif Multiple sequence alignment shaded by hydropathy between GP64 of Autographa californica multiple nucleopolyhedrovirus, Orgyia pseudotsugata multiple nucleopolyhedrovirus, *Thogotovirus thogotoense*, Apis thogotovirus 1, and Melitaea dydima thogotovirus 1.

Fig. S7 jvi.02148-24-s0007.tif Cryo-EM micrographs of budded viruses Ac-ATHOVGPgp64Δ.

Tables S1 and S2 jvi.02148-24-s0008.docx Sequences used for phylodynamic analysis and primers used for the amplification and molecular cloning of thogotovirus EFP genes.

Supplemental legends jvi.02148-24-s0009.docx Legends for Fig. S1 to S7.

ASM does not own the copyrights to Supplemental Material that may be linked to, or accessed through, an article. The authors have granted ASM a non-exclusive, world-wide license to publish the Supplemental Material files. Please contact the corresponding author directly for reuse.
