# Erratum for Milhomem Pilati Rodrigues et al., “Experimental and evolutionary evidence for horizontal transfer of an envelope fusion protein gene between thogotoviruses and baculoviruses”

**DOI:** 10.1128/jvi.01581-25

**Published:** 2025-10-08

**Authors:** Bruno Milhomem Pilati Rodrigues, Luis Janssen, Leonardo Assis da Silva, Suzane Suliane Vitorino Gomes Acacio, Mariana Tigano Magalhães, Bergmann Morais Ribeiro

## ERRATUM

Volume 99, no. 7, e02148-24, 2025, https://doi.org/10.1128/jvi.02148-24. Page 12, line 11: “positively charged” should read “negatively charged.”

