# Supplementary figures and images for: Experimental and evolutionary evidence for horizontal transfer of an envelope fusion protein gene between thogotoviruses and baculoviruses
Source: J Virol. 2025 Jun 25;99(7):e02148-24. doi: 10.1128/jvi.02148-24 (PMC12282062; doi:10.1128/jvi.02148-24)

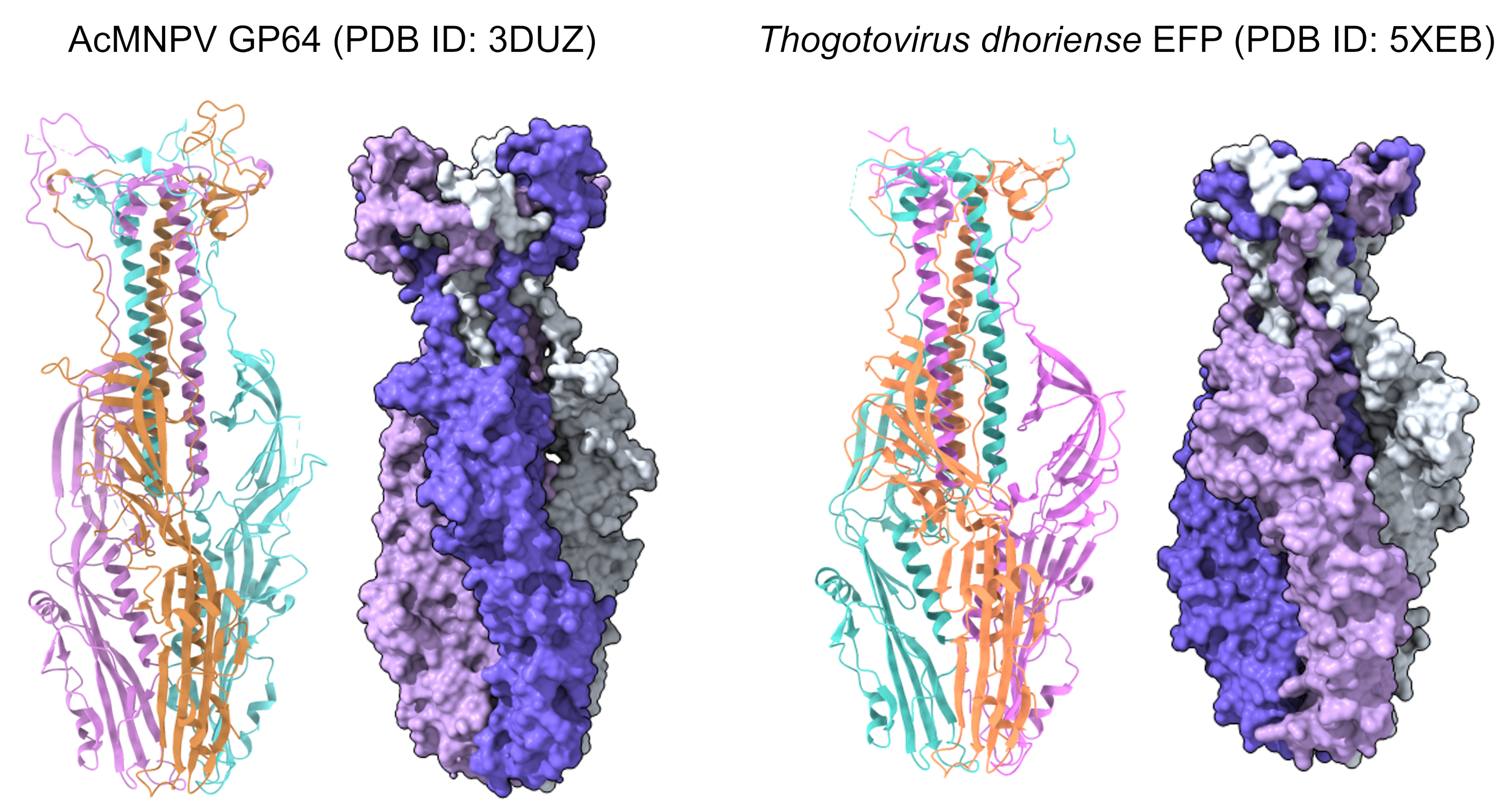

Supplement: Fig. S1 — Structural comparison of baculoviral GP64 from AcMNPV and thogotoviral EFP from Thogotovirus dhoriense. [file jvi.02148-24-s0001.tif]

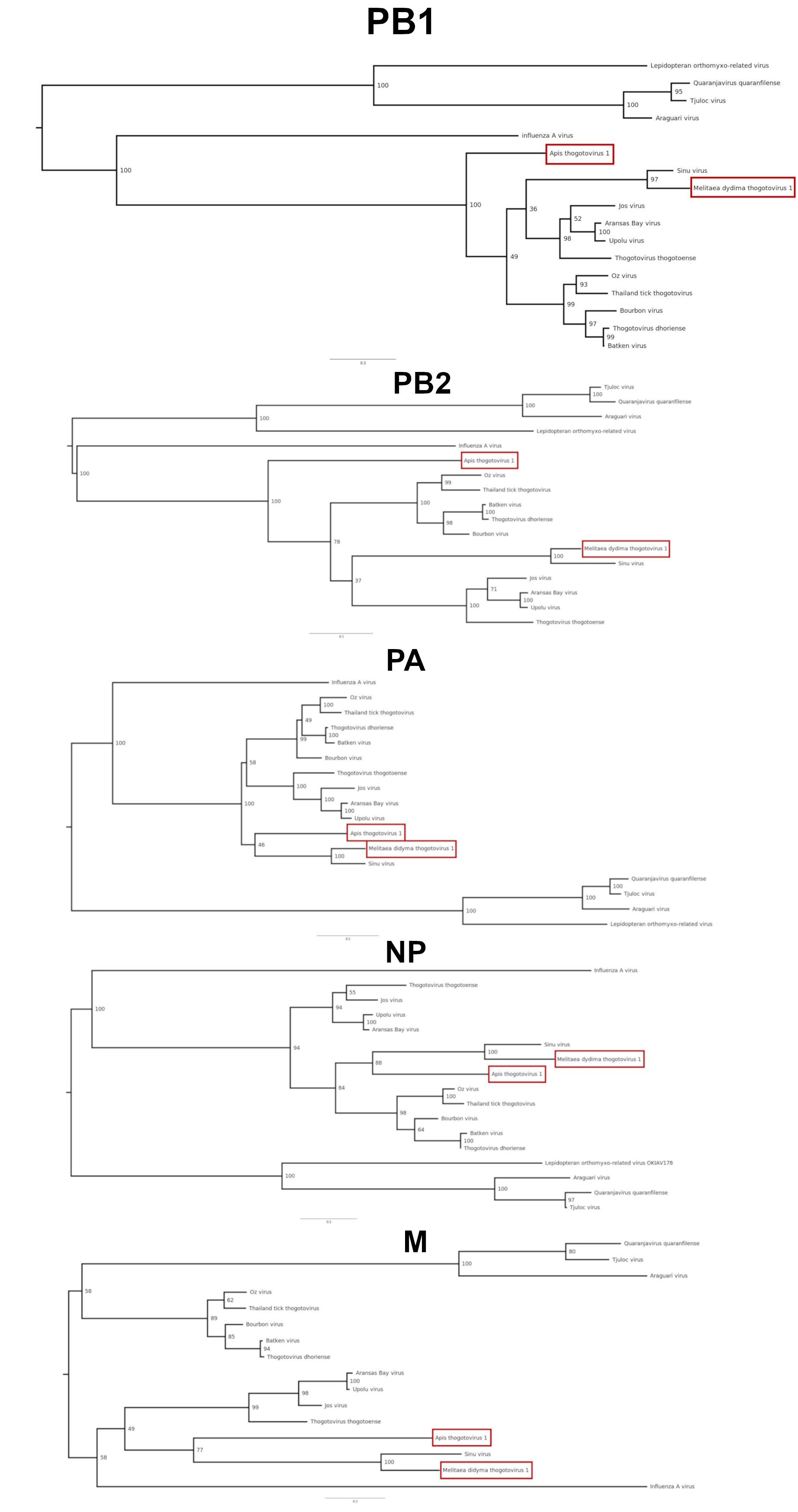

Supplement: Fig. S2 — Maximum likelihood phylogenies based on amino acid sequence of proteins from Melitaea didyma thogotovirus 1 and viral members of the Orthomyxoviridae, including ATHOV-1. [file jvi.02148-24-s0002.tif]

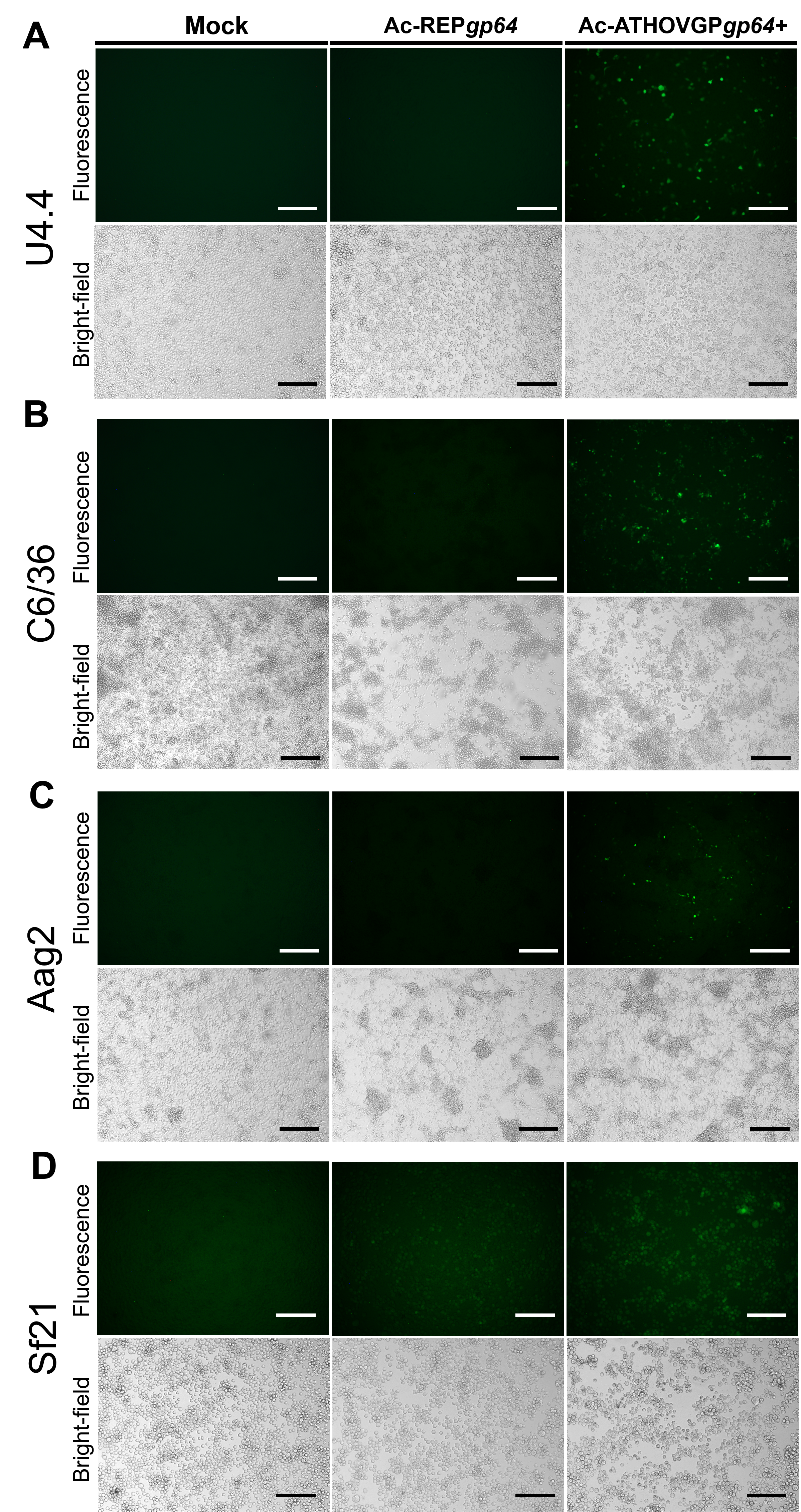

Supplement: Fig. S3 — Fluorescence microscopy of transduction in mosquito cell lines. [file jvi.02148-24-s0003.tif]

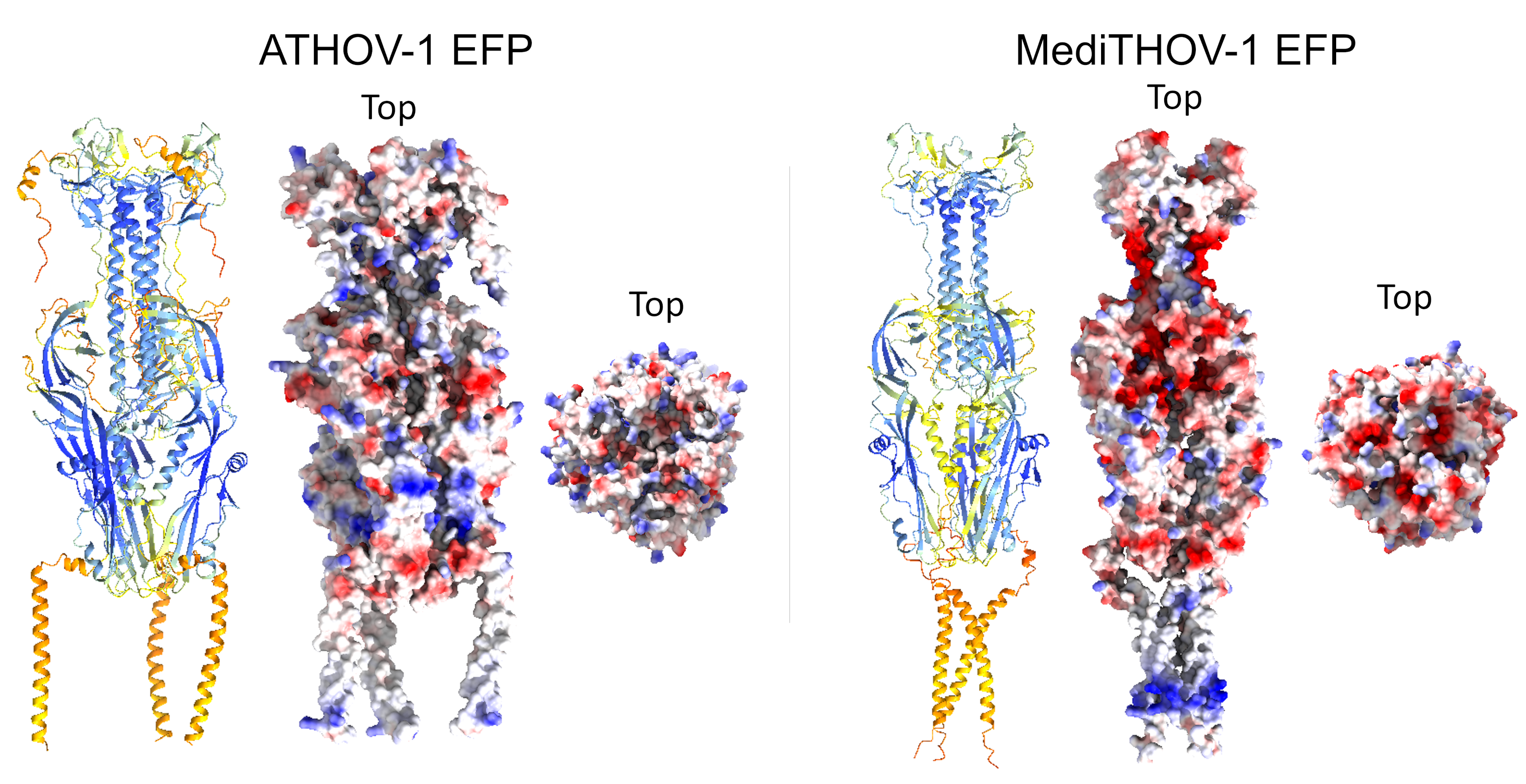

Supplement: Fig. S4 — Modeled structural comparison of EFPs from ATHOV-1 and MediTHOV-1. [file jvi.02148-24-s0004.tif]

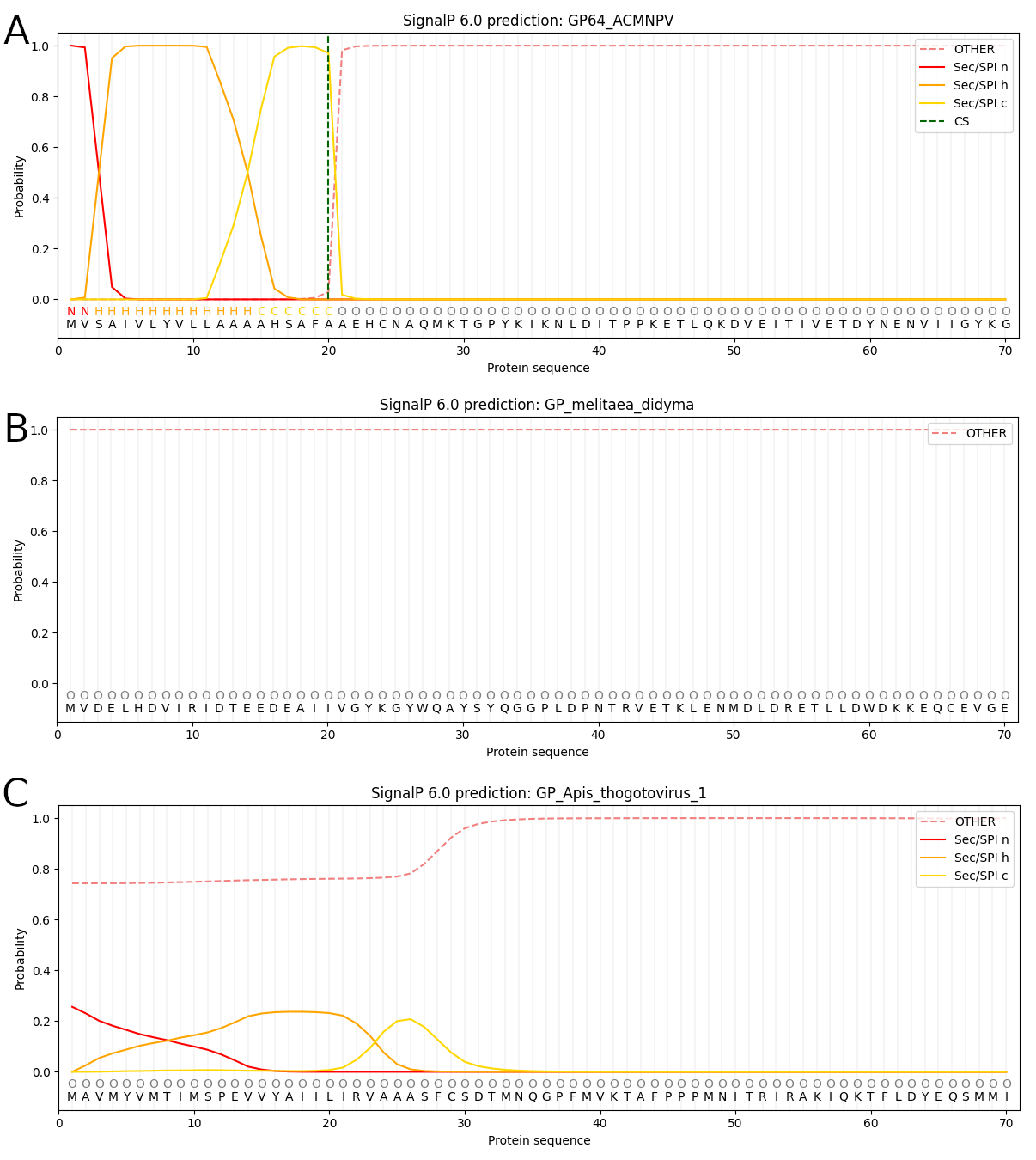

Supplement: Fig. S5 — SignalP prediction of signal peptides between the glycoproteins of AcMNPV, MediTHOV, and ATHOV-1. [file jvi.02148-24-s0005.tif]

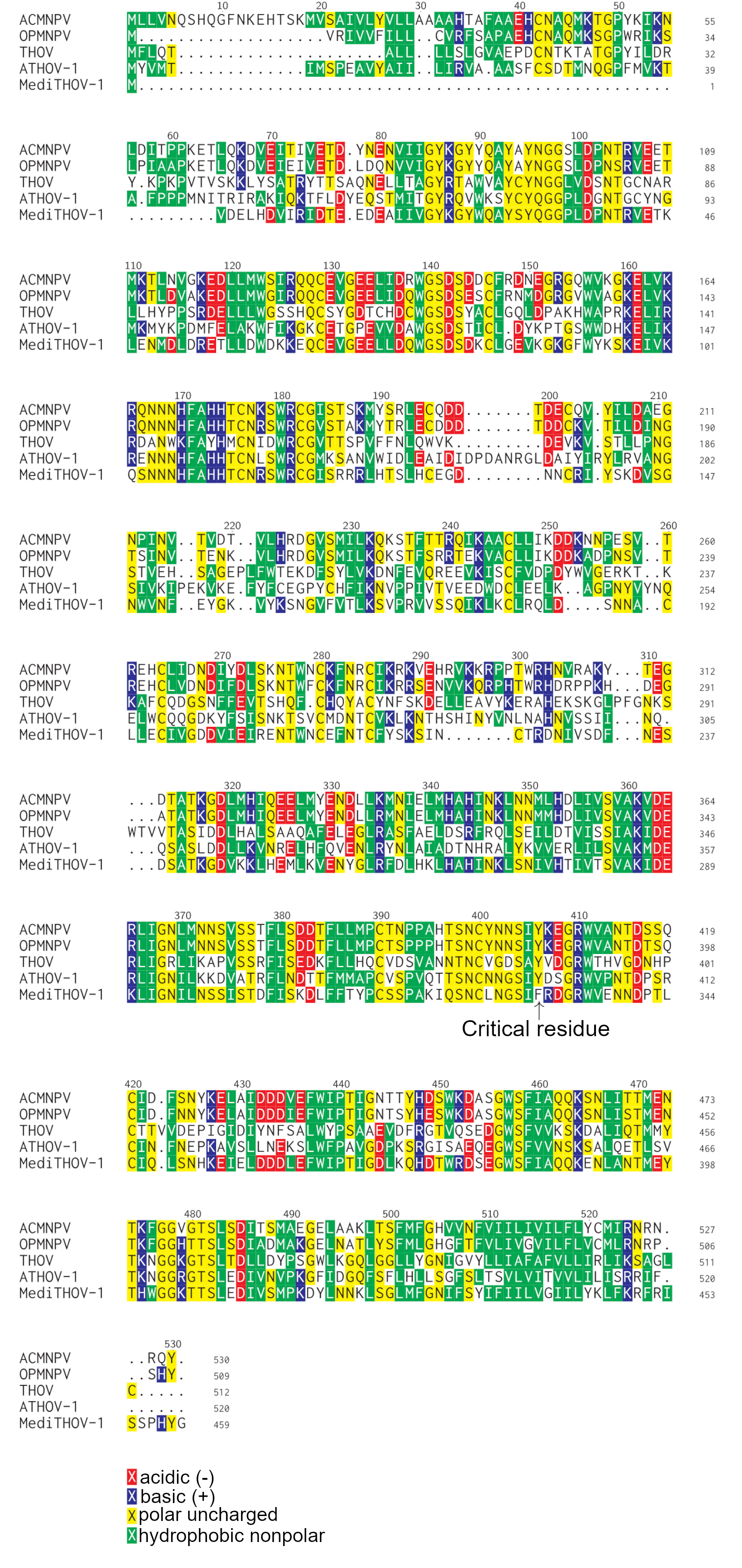

Supplement: Fig. S6 — Multiple sequence alignment shaded by hydropathy between GP64 of Autographa californica multiple nucleopolyhedrovirus, Orgyia pseudotsugata multiple nucleopolyhedrovirus, Thogotovirus thogotoense, Apis thogotovirus 1, and Melitaea dydima thogotovirus 1. [file jvi.02148-24-s0006.tif]

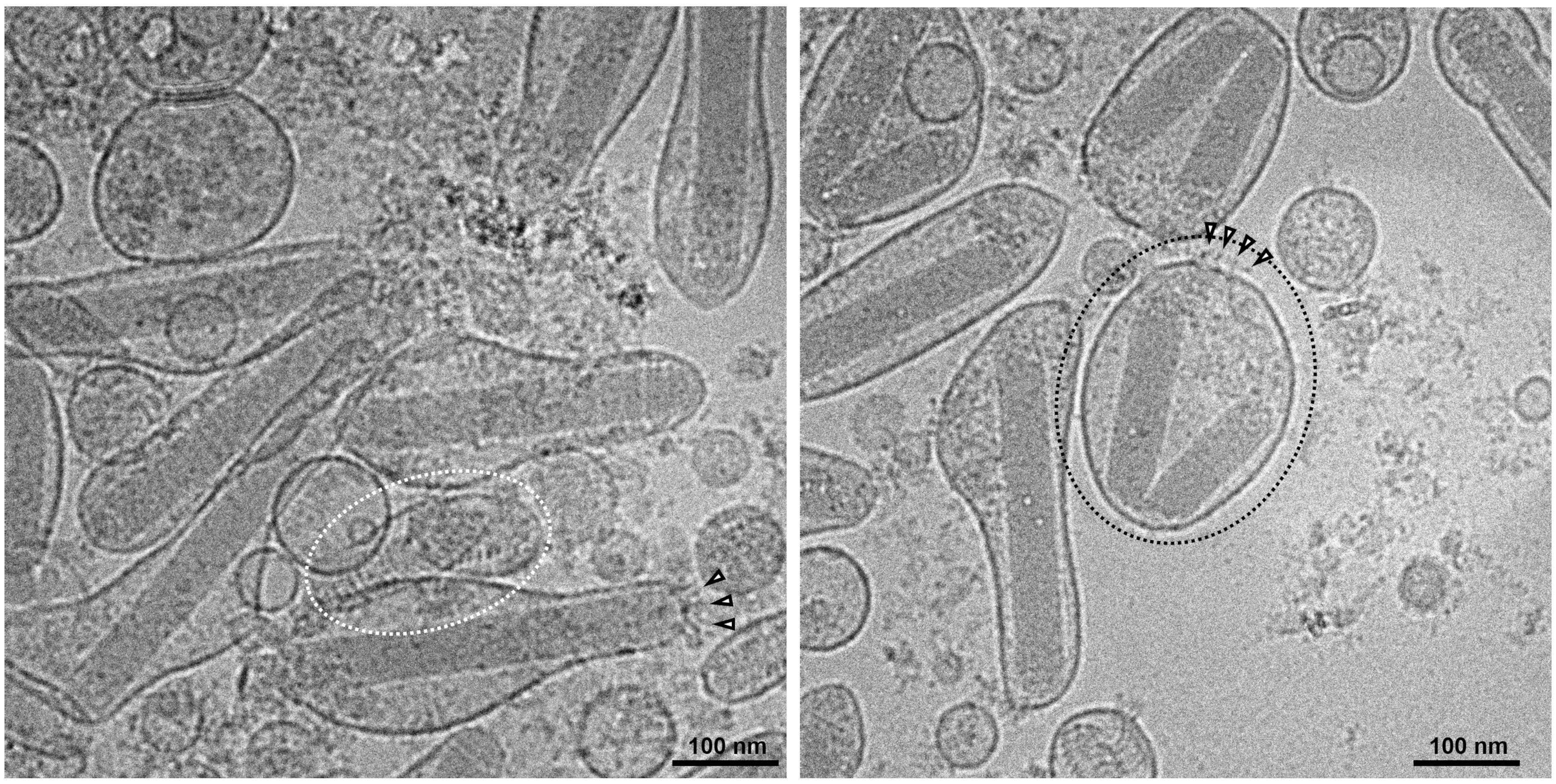

Supplement: Fig. S7 — Cryo-EM micrographs of budded viruses Ac-ATHOVGPgp64Δ. [file jvi.02148-24-s0007.tif]
