# Supplementary material for: Experimental and evolutionary evidence for horizontal transfer of an envelope fusion protein gene between thogotoviruses and baculoviruses
Source: J Virol. 2025 Jun 25;99(7):e02148-24. doi: 10.1128/jvi.02148-24 (PMC12282062; doi:10.1128/jvi.02148-24)
Supplement: Tables S1 and S2 — Sequences used for phylodynamic analysis and primers used for the amplification and molecular cloning of thogotovirus EFP genes. [file jvi.02148-24-s0008.docx]

**Table S1.** List of sequences used for phylodynamic analysis.

| **Virus** | **Acronym** | **Accession number** |
| --- | --- | --- |
| Dione juno nucleopolyhedrovirus | DJNPV | YP_010799788.1 |
| Hyphantria cunea nucleopolyhedrovirus | HCNPV | UIX56302.1 |
| Bombyx mori nucleopolyhedrovirus | BMNPV | QRM12746.1 |
| Catopsilia pomona nucleopolyhedrovirus | CPNPV | YP_009255283.1 |
| Troides aeacus nucleopolyhedrovirus | TANPV | QCF61100.2 |
| Lonomia obliqua multiple nucleopolyhedrovirus | LOMNPV | YP_009666390.1 |
| Neophasia sp. alphabaculovirus | NeNPV | QBC76009.1 |
| Antheraea proylei nucleopolyhedrovirus | APNPV | AYW35375.1 |
| Choristoneura rosaceana nucleopolyhedrovirus | CRNPV | YP_008378382.1 |
| Maruca vitrata nucleopolyhedrovirus | MVNPV | YP_950827.1 |
| Epiphyas postvittana nucleopolyhedrovirus | EPNPV | NP_203281.1 |
| Choristoneura occidentalis alphabaculovirus | CONPV | GR56918.1 |
| Parapoynx stagnalis nucleopolyhedrovirus | PSNPV | UZE89697.1 |
| Orgyia pseudotsugata multiple nucleopolyhedrovirus | OPMNPV | YP_046282.1 |
| Leucoma salicis nucleopolyhedrovirus | LSNPV | WZI19419.1 |
| Dasychira pudibunda nucleopolyhedrovirus | DPNPV | WHM28332.1 |
| Palpita vitrealis nucleopolyhedrovirus | PVNPV | USC25963.1 |
| Cyclophragma undans nucleopolyhedrovirus | CUNPV | YP_010086628.1 |
| Oxyplax ochracea nucleopolyhedrovirus | OONPV | YP_009666553.1 |
| Choristoneura murinana nucleopolyhedrovirus | CMNPV | YP_008992122.1 |
| Antheraea pernyi nucleopolyhedrovirus | ApeNPV | YP_611000.1 |
| Philosamia cynthia ricini nucleopolyhedrovirus virus | PCNPV | FY62837.1 |
| Anticarsia gemmatalis nucleopolyhedrovirus | AGNPV | AAM82816.1 |
| Choristoneura diversana nucleopolyhedrovirus | CDNPV | BU37509.1 |
| Thysanoplusia orichalcea nucleopolyhedrovirus | TONPV | YP_007250533.1 |
| Anagrapha falcifera MNPV | AFNPV | AAB53360.1 |
| Spilosoma obliqua nucleopolyhedrosis virus | SONPV | AUR45058.1 |
| Varroa orthomyxovirus-1 | VOTHOV | UDY81368.1 |
| Sinu virus | SINUV | APP91608.1 |
| Melitatea dydima orthomyxovirus 1 | MDTHOV | BK068798 |

**Table S2.** Primers used for the amplification and molecular cloning of thogotovirus EFP genes. Primer binding regions are highlighted in bold.

| Gene | Forward primer name/seq 5’>3’ | Reverse primer name/seq 5’>3’ | Product size |
| --- | --- | --- | --- |
| ATHOV-1 EFP | ATHOV-1-EFP_F/ TATGGGCCCA**ATGACCATTATGAGTCCCGAAGC** | ATHOV-1EFP_R/ CTAGCGGCCGCAGCTTAATGGTGATGGTGATGGTG**GAAAATTCTACGGGA** | 1,596 bp |
| MediTHOV-1 EFP | MediTHOV-1 EFP_F/ TATGGGCCCG**ATGGTTGATGAGCTCCATGA** | MediTHOV-1 EFP_R/ ATAGCGGCCGCTTAGTGATGGTGATGGTGATG**ACCATAATGGGGGGAGGATA** | 1,419 bp |
