# Supplementary material for: Experimental and evolutionary evidence for horizontal transfer of an envelope fusion protein gene between thogotoviruses and baculoviruses
Source: J Virol. 2025 Jun 25;99(7):e02148-24. doi: 10.1128/jvi.02148-24 (PMC12282062; doi:10.1128/jvi.02148-24)
Supplement: Supplemental legends — Legends for Fig. S1 to S7. [file jvi.02148-24-s0009.docx]

**Figure S1.** Structural comparison of baculoviral GP64 from AcMNPV and thogotoviral EFP from Thogotovirus dhoriense. Sctructures are show in ribbon representation and surface display collored by monomers that compose the homotrimers. Generated using ChimeraX v1.9.

**Figure S2.** Maximum likelihood phylogenies based on amino acid sequence of proteins from Melitaea didyma thogotovirus 1 and viral members of the Orthomyxoviridae, including ATHOV-1.

**Figure S3.** Fluorescence microscopy of transduction in mosquito cell lines. U4.4 cells (A), C6/36 cells (B), Aag2 cells (C), and Sf21 cells (D), inoculated at MOI 5 of Ac-REPgp64 and AcATHOVGPgp64+ viruses, and uninfected (mock) were observed by bright-field and fluorescence microscopy for the presence of the reporter gene eGFP at 48 hours post-infection (hpi). Scale bar: 250 μm.

**Figure S4.** Modelled structural comparison of EFPs from ATHOV-1 and MediTHOV-1. Ribbon representation colored by predicted local distance difference test (pLDDT), showing low confidence predictions regions in yellow and high quality prediction regions in blue. Surface representation colored by electrostatic charge, where blue is more positively charged and red negatively charged. Generated using ChimeraX v1.9.

**Figure S5.** SignalP prediction of signal peptides between the glycoproteins of **(A)** AcMNPV, **(B)** MediTHOV and **(C)** ATHOV-1.

**Figure S6.** Multiple sequence alignment shaded by hydropathy between GP64 of Autographa californica multiple nucleopolyhedrovirus, Orgyia pseudotsugata multiple nucleopolyhedrovirus, *Thogotovirus thogotoense*, Apis thogotovirus 1 and Melitaea dydima thogotovirus 1. A critical residue identified for cell fusion is highlighted with an arrow.

**Figure S7.** Cryo-EM micrographs of budded viruses Ac-ATHOVGPgp64Δ. White arrowheads indicate protruding EFP peplomers, dashed whit circle show a viral particle with relaxed form nuclecapsid and black dashed circle an enveloped viral particle containing two nucleocapsids.
